# Small-Scale Habitat Relationships of *Corydalus cornutus* Hellgrammites in Central Ohio Riffles

**DOI:** 10.3390/insects17040410

**Published:** 2026-04-10

**Authors:** Jon P. Bossley, Peter C. Smiley, Hanna E. Humphrey

**Affiliations:** 1 Division of Health and Life Science, College of Arts and Sciences, Mount Vernon Nazarene University, Mount Vernon, OH 43050, USA; hannahumphrey322@gmail.com; 2 USDA Agricultural Research Service, Columbus, OH 43210, USA; rocky.smiley@ars.usda.gov

**Keywords:** Megaloptera, water velocity, substrate size, spatial variables, mixed effects model analysis, rivers

## Abstract

*Corydalus cornutus* hellgrammites are known to inhabit riffles, but information is scarce regarding their habitat relationships at the plot scale and in the northern part of their range in the United States. We investigated the relationship of *C. cornutus* hellgrammite occurrence, density, and body size with environmental variables at the 1 m^2^ plot scale within central Ohio riffles. We collected hellgrammites and measured hydrological, substrate, large instream wood, and canopy cover variables in nine riffles in 2023 and ten riffles in 2024. Occurrence and density were best predicted by water velocity and grain size score, while none of the measured variables were a good predictor of head capsule width. Occurrence and density increased with increasing water velocity, grain size score, and substrate richness. Density also increased with increases in edge-interior scores. Head capsule width increased with increasing distances to the nearest plot with hellgrammites. Our results suggest that *C. cornutus* hellgrammites can serve as an indicator species because their occurrence and density reflect the water velocity and substrate conditions within riffles in the Midwestern United States.

## Simple Summary

Hellgrammites are large, aquatic larvae of the eastern dobsonfly. They live in riffles within mid-sized streams and rivers throughout eastern North America, where they function as predators of smaller insects and prey for sport fishes. Information regarding the environmental variables that influence hellgrammite occurrence, abundance and size is limited for their populations in the Midwestern United States. To address this issue, we conducted a two-year field study that documented the habitat relationships of hellgrammites within riffles in agricultural rivers in central Ohio during the early summer. Hellgrammite occurrence and abundance increased with water velocity, substrate size, and substrate diversity. Abundance increased in plots located in the center of the riffle, and body size increased with increasing distances to the nearest plot containing hellgrammites. Our results indicate that stream habitat management efforts that increase water velocity and substrate sizes within degraded riffles will benefit hellgrammites along agricultural rivers in the Midwestern United States.

## 1. Introduction

Megaloptera includes some of the world’s largest freshwater insects and consists of dobsonflies and alderflies (Corydalidae) along with fishflies (Sialidae). This insect order is comparatively small, having between 328 and 397 recognized extant species and sub-species that exhibit a patchy distribution globally [1,2]. The sub-family Corydalinae, which includes the largest species and is the focus of this paper, is found only in the Americas, Asia, and South Africa [1,2].

Development for all Megaloptera is holometabolous, involving an obligate aquatic larval stage. Larval Corydalinae in aquatic habitats respire via ventral tracheal gills and functional spiracles. Adult Corydalinae are known as dobsonflies while the larvae are referred to as hellgrammites. The larval body form is characterized by dorsoventral flattening, a leathery integument, lateral appendages, and powerful pincers. Hellgrammite development involves 11 instars and requires from one to three years to become winged adults pending latitude and temperature. *Corydalus cornutus* populations in the northern part of their range in the U.S. generally take longer to complete development than populations from the southern part of the U.S. range [3,4,5]. The impressive size associated with hellgrammites comes in the late instars with lengths up to 86 mm having been recorded for *C. cornutus* [6] and up to 90 mm for Corydalidae [7]. Total development from first to final instar may also involve an increase of up to 1000 times the original biomass [8].

Megaloptera have been used as indicator taxa within monitoring programs, but their assigned pollution sensitivity varies by the taxonomic level. At the family level, Corydalidae hellgrammites have been classified as pollution intolerant for water quality indices in the Midwestern United States [9,10]. *Corydalus cornutus* is classified as moderately intolerant by Ohio Environmental Protection Agency [11] and *Corydalus* spp. are recommended as indicator taxa for assessing the effects of pollutant mixtures and xenobiotics in streams and rivers in Mexico [12]. Corydalidae hellgrammites are also considered more sensitive pollution indicators than fishfly larvae [9]. It is well known that *C. cornutus* hellgrammites in the eastern United States are associated with stream riffles, which contain greater water velocities, higher oxygen levels, and greater food resources than other stream habitat types [4,13,14]. The association with riffles begins immediately after the first instar larvae hatch from their terrestrial egg cases, drop into the water, and then drift downstream until they encounter a suitable riffle [14]. Once established within the riffle, Corydalinae hellgrammites typically remain stationary but do exhibit limited nocturnal movement [2,15]. From the underside of rocks, hellgrammites engage in ambush predation on the aquatic larvae of other insects and occasionally smaller hellgrammites [13,15]. *Corydalus cornutus* hellgrammites’ preference for riffles and their absence from urban streams of central and southern Michigan [16] suggests that this species may be suitable for use as an indicator species for physical habitat conditions in streams.

Beyond the documented association of *C. cornutus* hellgrammites with riffles, there is limited information on their habitat use, especially in the northern portion of the range. Epperson and Short [4] documented that *C. cornutus* hellgrammites in 2nd and 4th order sites exhibited the greatest density in reaches having the greatest canopy cover, least discharge, and greatest amount of riffle habitat compared to larger downstream sites in the Guadalupe River, Texas. Short et al. [5] observed that the *C. cornutus* hellgrammite biomass and final instar lengths were greater in spring-fed than surface-fed streams and hellgrammite biomass increased with increasing wetted width in stream sites in the Guadalupe River Basin, Texas.

Location within the riffle also appears to be important to hellgrammites. Oviposition sites of *C. cornutus* dobsonflies most often occurred within the middle 1/3 of a sand-bottomed stream in Texas [14], likely to ensure that first instar hellgrammites are positioned over water upon hatching. Radio-tracking of Corydalinae hellgrammites in a stream in Japan also documented larval preference for the center of the riffle [15]. Substrate stability is another important determinant of position within the riffle. The turbulent hydrologic conditions within riffles likely draw *C. cornutus* hellgrammites to cobble and boulders, which provide more stable habitat due to their greater mass [16,17]. *Corydalus cornutus* hellgrammite biomass was best predicted by a combination of water depth, water velocity, and substrate size within riffles of a warmwater stream in Oklahoma [17]. Similarly, field studies in Arkansas and Utah streams documented that *Corydalus* hellgrammite density was greater within stable substrates (gravel, cobble, etc.) than on submerged and floating large instream wood [18,19]. However, field studies in low-gradient, sand-bottomed streams in Georgia, South Carolina, and Texas that lacked larger, stable substrate types showed that *C. cornutus* hellgrammite density and production were greater on large instream wood than on fine grain sediments (i.e., silt, clay, sand) [20,21,22].

Wherever they are positioned physically within the stream, *C. cornutus* hellgrammites occupy an important trophic position within lotic ecosystems, since they function as predators of invertebrates and prey for fishes [5,13,14,16,23]. Beyond threats posed by predation and other natural processes (i.e., floods, droughts), hellgrammites may also face anthropogenic threats from eutrophication, pollution, habitat alterations, and over-harvesting for use as bait [2,24]. Corydalinae are likely highly susceptible to habitat degradation due to their preference for riffle habitat and restricted instream movement as larvae, as well as their limited mobility as adults. Personal observations (J.P. Bossley) and anecdotal evidence from Ohio Environmental Protection Agency suggest *C. cornutus* hellgrammite populations in Ohio may be declining. Consequently, we investigated their habitat relationships within central Ohio riffles at the plot scale (1-m^2^) over a two-year period to help inform potential conservation strategies. We suspected that *C. cornutus* hellgrammites would preferentially occur in plots containing larger substrate (i.e., cobble and boulders) and faster water velocities. Specifically, our research questions were: (1) What environmental variables are good predictors of *C. cornutus* hellgrammite occurrence, density, and body size at the plot scale within riffles in central Ohio rivers; and (2) What is the relationship of *C. cornutus* hellgrammite occurrence, density, and body size with environmental variables at the plot scale within riffles in central Ohio rivers?

## 2. Materials and Methods

### 2.1. Study Area

**Figure 1:**
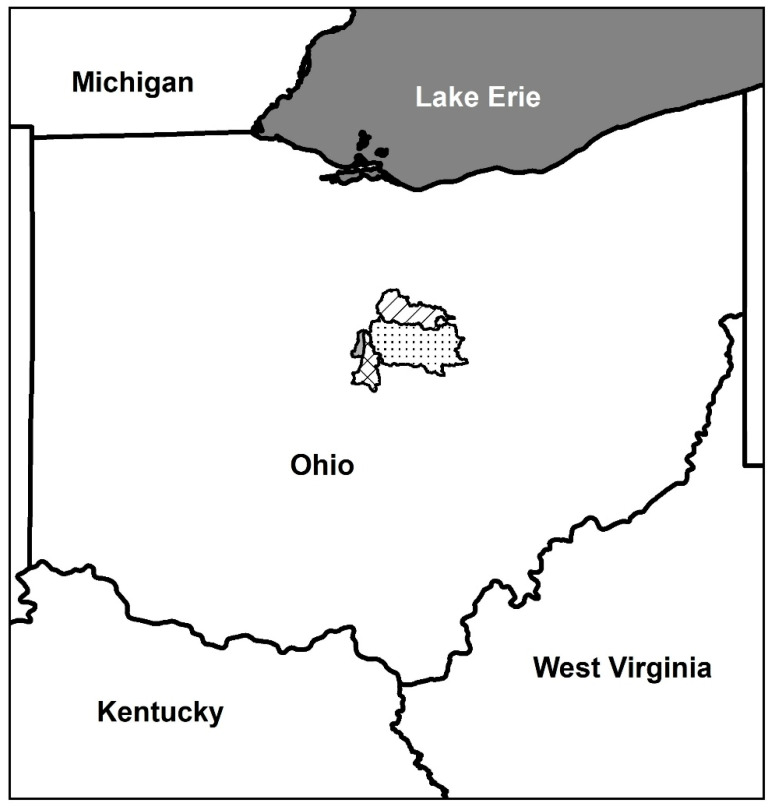
Map depicting the locations of the Alum Creek watershed (solid gray), the Upper Big Walnut Creek watershed (cross hatch), the Kokosing River watershed (simple stipple), and the Clear Fork River watershed (diagonal lines) in Ohio, United States.

We selected 10 riffles in four river basins in central Ohio as study sites (Figure 1, Supplementary File S1). Selected riffles were those occurring in fourth- or fifth-order rivers within a 43 km radius of Mount Vernon Nazarene University that represented a range of adjacent land use conditions. Specifically, we sampled two riffles in Alum Creek, two riffles in the Upper Big Walnut Creek, three riffles in the Clear Fork River, two riffles in the Kokosing River, and one riffle in a tributary of the Kokosing River (Jelloway Creek). Alum Creek and Upper Big Walnut Creek are part of the Scioto River Basin, while Clear Fork River, Kokosing River, and Jelloway Creek are all part of the Muskingum River Basin. Both Clear Fork River and Kokosing River are state scenic rivers. Land use within the watersheds of Alum Creek, Upper Big Walnut Creek, and Kokosing River is mostly agriculture (54.6 to 64.4%), while land use in the Clear Fork River watershed is dominated by both forest (42.2 to 42.6%) and agriculture (44.8 to 45.2%) (Table 1). The watershed sizes of our selected riffles ranged from 65 to 1226 km^2^ (Table 1) and all riffles are comprised of primarily sedimentary substrate (i.e., sandstone and shale). Preliminary nutrient measurements in 2024 and 2025 indicate that average nutrient levels in our study sites are below the U.S. Environmental Protection Agency drinking water standards and those expected to cause harmful algal blooms (J.P. Bossley, unpublished data).

We sampled hellgrammites and measured habitat variables in nine riffles in 2023 and 10 riffles in 2024. Sampling was conducted between 5 June 2023 to 19 July 2023 and 4 June 2024 to 9 July 2024. Our sampling occurred during the early summer, which is the time period when *C. cornutus* adults begin to emerge. During each sampling visit, we delineated the upstream and downstream boundaries of each riffle, measured the riffle length, and then established three transects located near the upstream border, middle, and downstream border of the riffle. The distance between transects depended on the riffle length and ranged from 7.6 to 36.2 m in riffles ranging in length from 18.3 to 70.3 m. After establishing the transects, we obtained the wetted width of each transect, and wetted widths ranged from 10.6 to 40.3 m (Table 1). Additionally, discharge during sampling ranged from 0.33 m^3^/s to 4.68 m^3^/s (Table 1).

We used information from the measured wetted widths to establish five systematic 1 m by 1 m plots located equidistantly along each transect. Distance between systematic plots along each of the three transects ranged from 1.07 to 7.11 m and was dependent on the wetted width of the transect. We also sampled five additional subjective plots that were selected because these were locations in the riffle that contained turbulent water flow and boulder and cobble substrates, which we suspected would be good hellgrammite habitats. Distances between the subjective plots ranged from 1.28 to 25.40 m. In summary, we sampled 180 plots in nine riffles in 2023 and 200 plots in 10 riffles in 2024.

### 2.2. Hellgrammite Sampling

We sampled hellgrammites from 20 1-m^2^ plots in each riffle. Specifically, sampling for hellgrammites was conducted with a d-frame dipnet (width of dipnet mouth–32 cm; height of dipnet mouth–26 cm; mesh size–500 microns size). Sampling involved placing the dipnet at the right downstream boundary of a 1 m × 1 m plot and then disturbing all substrate upstream for one minute while moving laterally across the full length of the plot. If we reached the left boundary of the plot before 1 min, we then reversed direction and continued sampling for the remainder of the allotted time. Doing so enabled us to ensure that sampling time within each plot was equal. Captured hellgrammites were then placed in a pan, identified, measured for body length and head capsule width, enumerated, and then released alive back into the riffle. Hellgrammite body lengths and head capsule widths were measured to the nearest mm with a ruler.

### 2.3. Physical, Spatial, and Chemical Variable Measurements

We measured hydrologic variables and estimated canopy cover, large instream wood, and substrate in each plot sampled in 2023 and 2024. A single measurement of water depth and water velocity was obtained from the center of each 1 m^2^ plot with a top setting wading rod and an electromagnetic velocity meter. Riparian canopy cover above each plot was visually estimated. Within each plot we noted the occurrence of large instream wood (i.e., logs, root wads, or accumulations) and estimated the percentages of boulder, cobble, gravel, fines (i.e., combined sand, silt, and clay), and bedrock. Substrate information was used to calculate substrate richness (the number of substrate types in a plot) and to identify the dominant substrate type within each plot. Information on the dominant substrate type enabled us to calculate the percentage of cobble and boulders in each plot and the dominant grain size scores for each plot. Dominant grain size score was calculated by first determining the dominant substrate type for each plot and then assigning a grain size value. Grain size values were assigned accordingly: bedrock–0 mm; fines–1.00 mm; gravel–33 mm; cobble–160 mm; boulder–260 mm. Assigned grain size scores for fines, gravel, and cobble were the median values of the range of Wentworth grain size scores for each of these substrate types [28]. The assigned score for boulder was the minimum value within its Wentworth size class (256 mm) rounded up to the nearest tenth and the score for bedrock was zero because it is not sediment and does not have a Wentworth grain score. If a plot was dominated by two substrate types, then the assigned grain size score for the plot was the mean grain size value of the two substrate types.

We measured the distances of each plot from the left water’s edge and to the downstream boundary of the riffle. This spatial information was used in conjunction with information on hellgrammite occurrence to calculate the distance of each plot to the nearest plot with hellgrammites in each riffle and the distance of each plot to the centroid of all plots with hellgrammites within each riffle. The distances of each plot to all other plots in a site were calculated using the dist function from the stats package [29]. Spatial information was also used to calculate edge-interior scores. We developed this novel index for our analysis, and it reflects the position of the plot within the riffles to help us determine if *C. cornutus* hellgrammites prefer plots within the center of the riffle or those plots near the water’s edge and/or upstream and downstream riffle boundaries. To calculate edge-interior scores, we divided the distance of the plot from the left water’s edge by the wetted width of the nearest transect, which expresses the plot position as a proportion of the wetted width. We also divided the distance of the plot from the downstream riffle boundary by the riffle length, which expresses the plot position as a proportion of the riffle length. We assigned the scores for each proportion of the wetted width (*x*-axis) and proportion of riffle length (*y*-axis) as follows: 0.165 (0.000–0.199 and 0.800–1.000); 0.333 (0.200–0.399 and 0.600–0.799); 0.500 (0.400–0.599). Finally, edge-interior scores were calculated as the sum of the assigned score for proportion of wetted width and assigned score for proportion of riffle length. Edge-interior scores ranged from a minimum of 0.33 for plots located near the water’s edge and the upstream or downstream riffle border to a maximum of 1.00 for plots located in the center of the riffle.

### 2.4. Statistical Analyses

All statistical analyses were conducted with R version 4.4.1 [29] and a significance level of 0.05. *Corydalus cornutus* hellgrammite occurrence, mean density, and mean head capsule width were calculated for each plot sampled and served as our response variables in all statistical analyses. Additionally, we chose 10 habitat variables for our statistical analyses that consisted of water depth, water velocity, substrate richness, percent boulders and cobble, grain size score, large instream wood occurrence, percent canopy cover, edge-interior scores, distance to nearest plot with hellgrammites, and distance to centroid of all plots with hellgrammites. All statistical analyses involved mixed effects analysis because we repeatedly sampled the same riffles in the same rivers for a two-year period.

We conducted mixed effects model regression tree analysis with all 10 habitat variables as the fixed effects and sites nested within watersheds as the random effect to identify the best predictor of the three hellgrammite response variables. Residuals from occurrence models were not expected to meet the parametric assumptions of normality and homogeneity and preliminary analyses indicated that the residuals from an initial density model did not meet the parametric assumptions. Subsequently, we conducted generalized linear mixed effects model regression tree analysis using the binomial family for occurrence and the Poisson family for mean density. Preliminary analyses also indicated that mean head capsule width models did not meet the parametric assumptions, but mean head capsule width is a measurement that does not yield count data or whole number values. Therefore, we used log x + 1 transformed head capsule widths and conducted linear mixed effects model regression tree analyses. We also conducted mixed effects model regression tree analysis with no fixed effects and sites nested within watersheds as the random effect and we compared the AIC scores from these null model regression trees (intercept only regression trees) with the resulting regression trees produced with the 10 fixed effects. Generalized linear mixed effects model regression tree analysis was conducted with the glmertree function from the glmertree package [30] and linear mixed effects model regression tree analysis was conducted with the lmertree function, which is also part of the glmertree package. AIC values were obtained with the AIC function from the stats package [29].

We also conducted linear and generalized linear mixed effects model analysis with a subset of seven fixed effects that did not exhibit moderate to strong multicollinearity (|r| ≥ 0.50). The subset of habitat variables used as fixed effects in this analysis included water depth, water velocity, substrate richness, grain size score, percent canopy cover, edge-interior scores, and distance to nearest hellgrammite plot. For these analyses we wanted to determine the relationships of the three hellgrammite response variables with the seven fixed effects using a multiple variable model that would depict the effect of each variable in the model while holding the effects of the other variables in the model constant. However, we were also concerned about the potential for overfitting the models by having too many fixed effects. Therefore, we developed an initial two variable model with water velocity and grain size score, which were the two fixed effects identified as the best predictors of occurrence and density in the mixed effects regression tree results (see details below in Results). We then created a three-variable model by adding one of the other five fixed effects and then examining the *p* values of the added fixed effect. If the added fixed effect was not significant (*p* > 0.05), then it was excluded from further analyses. If the added fixed effect was significant (*p* < 0.05), then it was retained for our final analyses. This screening process resulted in the final models for occurrence and density containing the fixed effects of water velocity, grain size score, substrate richness, and edge-interior score. The final model for log transformed head capsule width contained water velocity, grain size score, and distance to nearest hellgrammite plot. All generalized linear mixed effect model analyses and linear regression analyses were conducted using glmmTMB function from the package glmmTMB [31]. We specified the binomial family for occurrence, the negative binomial family for density, and the Gaussian family for log transformed head capsule width. For occurrence and density, the random effect was site nested within watersheds. However, model convergence issues occurred with our head capsule width models that were resolved by dropping the random effect. Examination of the random effect terms in the initial head capsule width models indicated that the random effects had little or no effect and subsequent comparisons of AIC scores confirmed that dropping the random effect term improved these models.

## 3. Results

We captured 276 hellgrammites over two years of sampling, and all were identified as eastern dobsonfly (*C. cornutus*). Captured hellgrammites ranged in body length from 11 to 90 mm and the head capsule widths of captured individuals ranged from 1 to 11 mm. Simple linear regression analyses confirmed that head capsule width was strongly (adjusted R^2^ = 0.888) and positively correlated (*p* < 0.001) with body length, which supported our use of head capsule width as proxy for body size. In 2023 and 2024 combined, 101 plots (26.6%) sampled contained hellgrammites and 279 plots (73.4%) did not. Hellgrammite density within plots ranged from 0 to 11 individuals/m^2^ with an overall average density of 0.74 individuals/m^2^ during the two years of our sampling. Mean hellgrammite head capsule width within plots ranged from 1.50 to 10.00 mm with an overall average of 4.79 mm for the two-year period.

Water depth within the plots ranged from 0.03 to 0.70 m with an average of 0.20 m and water velocity within the plots ranged from −0.02 to 1.27 m/s with an average of 0.41 m/s. Grain size scores within the plots ranged from 0 (bedrock dominated) to 260 (boulder dominated) with an average grain size score of 149, which is slightly smaller than cobble. Large instream wood was rare in our study plots and only occurred in 2 of 380 plots sampled. Qualitative observations indicated that leaf packs and other organic matter accumulations occurred infrequently within our sampled plots. Visual estimates of percent canopy cover over the plots ranged from 0 to 100% with an average of 32%. Distance to the nearest plot with hellgrammites ranged from 0.53 to 33.5 m with an average of 7.28 m.

**Figure 2:**
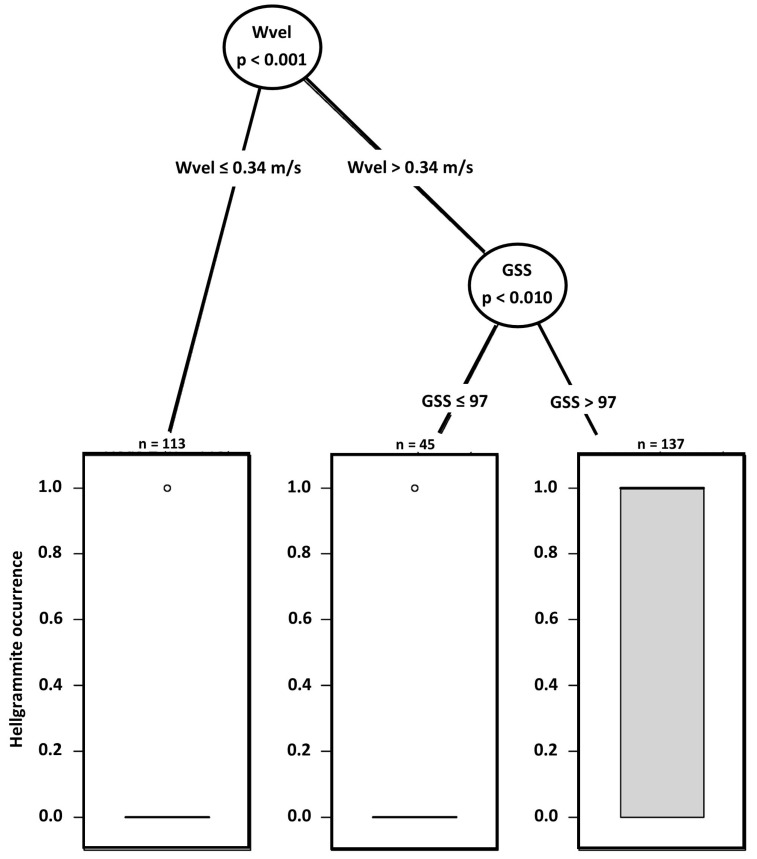
Generalized linear mixed effects model regression tree for *Corydalus cornutus* hellgrammite occurrence in riffles within Alum Creek, Upper Big Walnut Creek, Kokosing River, and Clear Fork River, Ohio, 2023–2024. Abbreviations are: Wvel—water velocity; GSS—grain size score.

**Figure 3:**
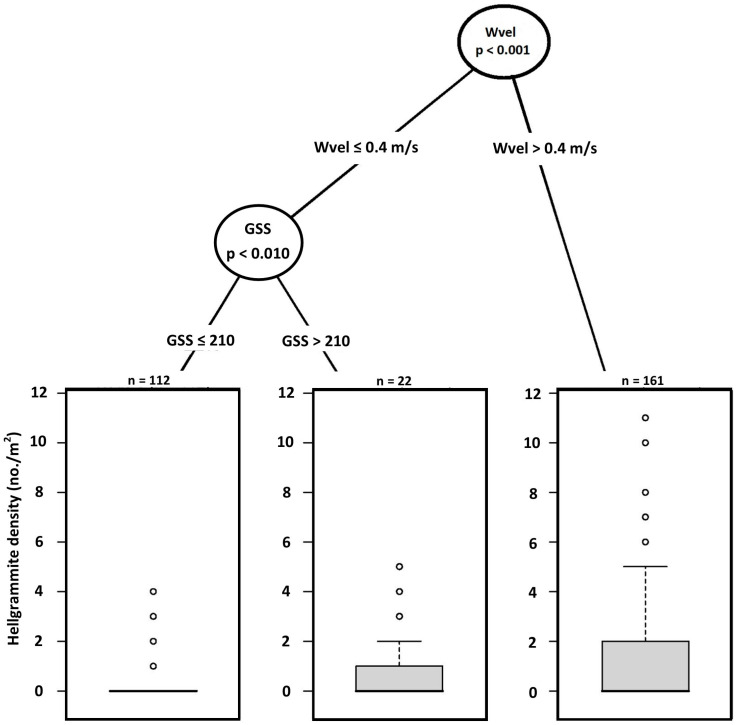
Generalized linear mixed effects model regression tree for *Corydalus cornutus* hellgrammite density in riffles within Alum Creek, Upper Big Walnut Creek, Kokosing River, and Clear Fork River, Ohio, 2023–2024. Abbreviations are: Wvel—water velocity; GSS—grain size score.

Mixed effects regression tree analyses indicated that hellgrammite occurrence in riffles was predicted by water velocity and grain size score where the greatest occurrence was in plots with water velocities greater than 0.34 m/s and grain size scores greater than 97 (Figure 2). The lowest occurrence was in plots with water velocities greater than 0.34 m/s and grain size scores less than or equal to 97 and plots with water velocities less than or equal to 0.34 m/s (Figure 2). AIC comparisons indicated that the mixed effects regression tree for occurrence (AIC = 231.62) was better than the null occurrence mixed effects regression tree (AIC = 296.82). Hellgrammite density in riffles was also predicted by water velocity and grain size score where the greatest density occurred in plots having water velocities greater than 0.40 m/s (Figure 3). Plots having water velocities less than or equal to 0.40 m/s and grain size scores greater than 210 had greater density than plots with water velocities less than or equal to 0.40 m/s and grain size scores less than or equal to 210 (Figure 3). AIC comparisons indicated that the mixed effects regression tree for density (AIC = 635.17) was better than the null density mixed effects regression tree (AIC = 748.60). The linear mixed effects regression tree of hellgrammite head capsule width did not produce a regression tree as none of the 10 measured habitat variables was a significant predictor of head capsule width.

**Figure 4:**
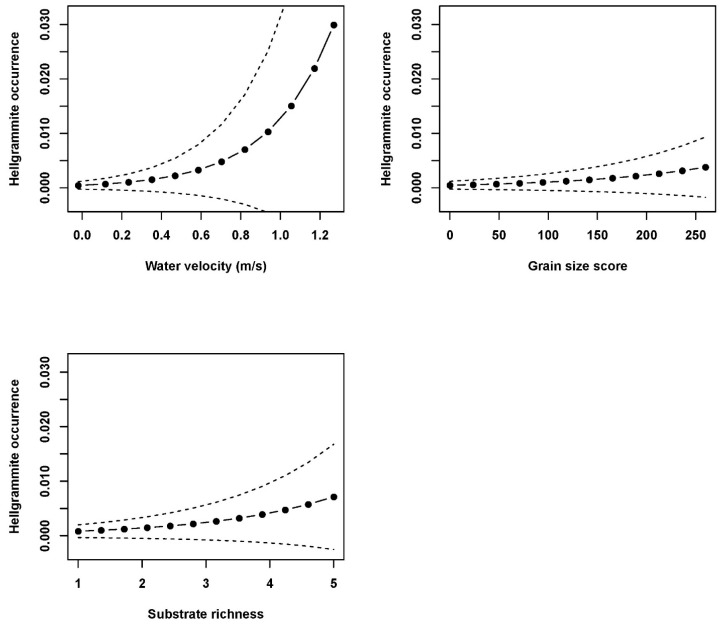
Predicted generalized linear mixed effects model relationships of *Corydalus cornutus* hellgrammite occurrence with water velocity, grain size score, and substrate richness in riffles within Alum Creek, Upper Big Walnut Creek, Kokosing River, and Clear Fork River, Ohio, 2023–2024. Each subfigure depicts the relationship of hellgrammite occurrence with the specified fixed effect and all other fixed effects held constant. The solid line with circles depicts the predicted relationship and the dashed lines signify the standard error of the predicted relationship.

**Figure 5:**
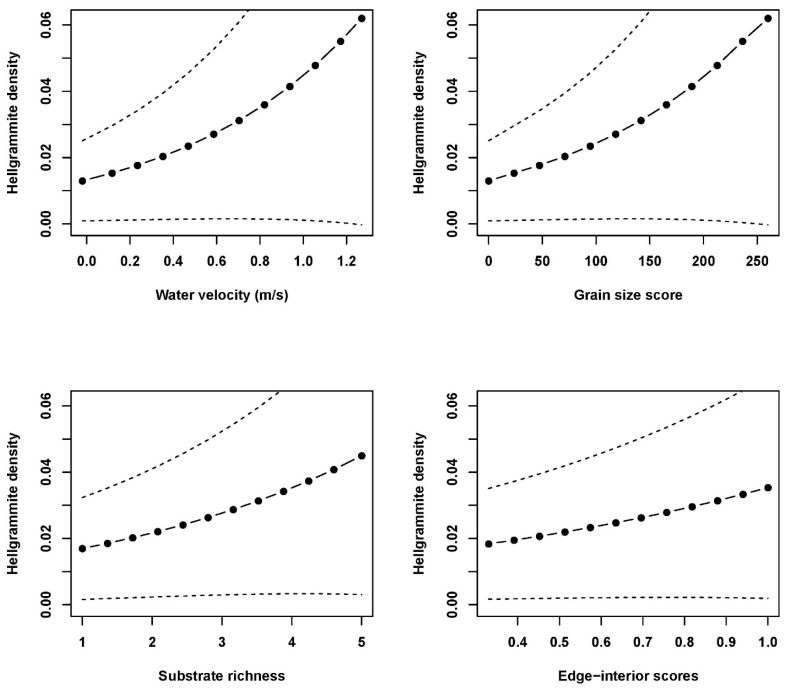
Predicted generalized linear mixed effects model relationships of *Corydalus cornutus* hellgrammite density with water velocity, grain size score, substrate richness, and edge-interior scores in riffles within Alum Creek, Upper Big Walnut Creek, Kokosing River, and Clear Fork River, Ohio, 2023–2024. Each subfigure depicts the relationship of hellgrammite density with the specified fixed effect with all other fixed effects held constant. The solid line with circles depicts the predicted relationship and the dashed lines signify the standard error of the predicted relationship.

**Figure 6:**
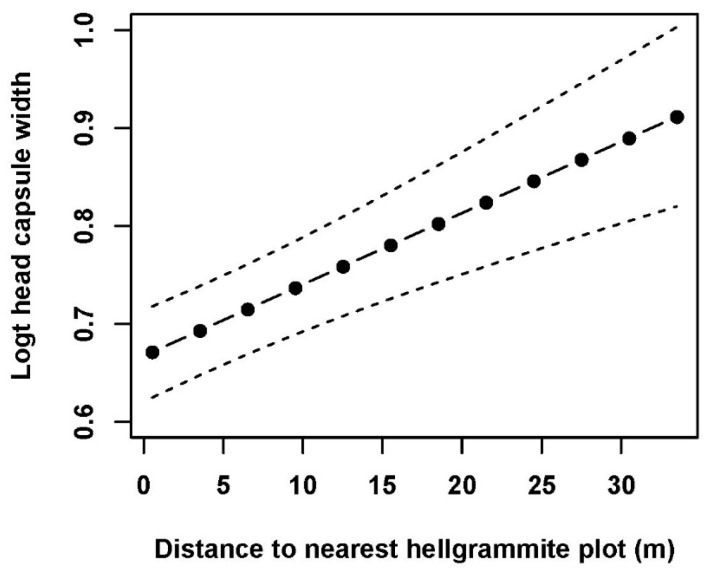
Predicted generalized linear mixed effects model relationships of log(x + 1) transformed (Logt) *Corydalus cornutus* hellgrammite head capsule width with distance to nearest hellgrammite plot in riffles within Alum Creek, Upper Big Walnut Creek, Kokosing River, and Clear Fork River, Ohio, 2023–2024. This figure depicts the relationship of hellgrammite head capsule width with the distance to nearest hellgrammite plot and water velocity and grain size score held constant. The solid line with circles depicts the predicted relationship and the dashed lines signify the standard error of the predicted relationship.

Mixed effects model analyses with the subset of measured environmental variables indicated that hellgrammite occurrence in riffles was significantly correlated with water velocity, grain size score, and substrate richness and not significantly correlated with edge-interior scores (Table 2). Occurrence increased with increasing water velocity, grain size score, and substrate richness within plots in riffles (Figure 4). Hellgrammite density in riffles was significantly correlated with water velocity, grain size score, substrate richness, and edge-interior scores and density increased with increases in water velocity, grain size score, substrate richness, and edge-interior scores (Figure 5). Hellgrammite head capsule width was significantly correlated with distance to nearest plot with hellgrammites and was not significantly correlated with water velocity and grain size score (Table 2). Head capsule width increased with increasing distances to the nearest plot with hellgrammites (Figure 6).

## 4. Discussion

Our results indicated that the best predictors of *C. cornutus* hellgrammite occurrence and density at the 1 m^2^ plot scale within riffles of central Ohio rivers were water velocity and grain size score. None of the measured environmental variables were found to be a good predictor of *C. cornutus* hellgrammite head capsule width. *Corydalus cornutus* hellgrammite population characteristics at the 1 m^2^ plot scale within riffles of central Ohio rivers were also positively correlated with water velocity, grain size score, substrate richness, edge-interior scores, and distance to the nearest plot with hellgrammites.

Our findings related to the importance of water velocity and grain size score aligned with our expectations of *C. cornutus* hellgrammite habitat relationships. These findings are also consistent with *C. cornutus* hellgrammite preferences for riffle habitats that are characterized by the combination of fast water velocities and large substrate sizes [4,13,14]. Our results are similar to those of Orth and Maughan [17] who documented that *C. cornutus* hellgrammite biomass in riffles in a warmwater stream in Oklahoma was best predicted by a combination of water depth, velocity, and substrate type. However, we documented an interaction effect of water velocity and grain size scores on *C. cornutus* hellgrammite occurrence and density. Specifically, water velocity was the primary determinant of *C. cornutus* occurrence and density within 1-m^2^ plots within riffles and grain size score was a secondary factor that influenced occurrence in plots with water velocities >0.34 m/s and density in plots with water velocities ≤0.40 m/s. To our knowledge we are the first to document the influence of an interaction effect of water velocity and grain size score on *C. cornutus* hellgrammites. 

Our results at the plot scale combined with those at the reach scale [4,13,14] indicate *C. cornutus* hellgrammites’ preference for fast water velocities and large substrate sizes in streams and rivers at both the plot scale within individual riffles and at the reach scale encompassing multiple habitat types (pools, riffles, runs, etc.). The preference of *C. cornutus* hellgrammites for riffle habitat in the eastern U.S. has been attributed to these habitats containing greater streamflow, higher oxygen levels, and greater food resources than other habitat types [4,13,14]. It is likely that the preference of *C. cornutus* hellgrammites for fast water velocities and large substrate sizes at the plot scale also corresponds to increased oxygen levels and greater food resources, but this needs to be confirmed by future research.

We also documented that *C. cornutus* hellgrammite occurrence and density increased with increasing substrate richness within the plots. We believe our study is the first to document a relationship between *C. cornutus* hellgrammite occurrence and density with this substrate variable. We originally thought that this relationship might be influenced by the underlying effect of the larger substrates (i.e., cobble and boulder) on *C. cornutus* hellgrammite occurrence and density, but substrate richness was not correlated (*p* < 0.05) with percent cobble and boulders, grain size score, or any other habitat variable. The observed relationship between *C. cornutus* hellgrammite occurrence and density with substrate richness might reflect the ability of a greater assortment of substrate sizes to provide shelter for a greater size range of *C. cornutus* hellgrammites or it might reflect the effect of an unmeasured habitat variable. We recommend that future research explore the specific mechanisms underlying the influence of substrate richness on *C. cornutus* hellgrammites.

We observed that *C. cornutus* hellgrammite density increased with increasing edge- interior scores, which signifies that density increased in plots located in the center of the riffle compared to plots located at the periphery of the riffle. These results are concordant with those of Lowery and Cook [14] and Hayashi and Nakane [15]. In a sand-bottomed stream in Texas, *C. cornutus* dobsonflies most often selected oviposition sites in the middle 1/3 of the stream [14], which would increase the likelihood that newly hatched hellgrammites would drop into the water rather than the dry streambank. Hayashi and Nakane [15] radio-tagged final instar *Protohermes grandis* hellgrammites (Cordalinae) and documented that newly released *Protohermes grandis* hellgrammites migrated from the water’s edge to the center of the riffle within a stream in Japan. Along with these findings, our results suggest that the preference for the center of a riffle is reflected in both adult oviposition behavior and hellgrammite habitat selection behavior. Regarding habitat selection behavior, the causal factors for Corydalinae hellgrammites’ preference for the center of the riffle remain unknown. Future research is needed to determine whether this preference occurs primarily due to the water velocity and substrate conditions or because the center of the riffle provides an ideal position for ambush predation [15] or a combination of both factors.

*Corydalus cornutus* head capsule width, which is an indicator of body size, increased with increasing distances to the nearest plot with *C. cornutus* hellgrammites within central Ohio riffles during the early summer. Laboratory experiments with large and small *C. cornutus* hellgrammites found that larger individuals often excluded smaller individuals from refugia [23], and such exclusionary behavior may be related to the known tendency of Megaloptera larvae to engage in cannibalism [1,2]. Stewart et al. [13] confirmed the cannibalistic tendencies of *C. cornutus* hellgrammites by examining the stomach contents of hellgrammites from a large riffle in a Texas River and found that *C. cornutus* hellgrammites constituted 0.4% of the food items found in stomachs of *C. cornutus* hellgrammites having head capsule widths between 6 and 8 mm. Thus, the positive association between *C. cornutus* hellgrammite head capsule widths and increasing distances from nearest plot with hellgrammites likely reflects a combination of the territorial nature of the larger hellgrammites and the risk of cannibalism. 

Notably, *C. cornutus* hellgrammite head capsule width was not correlated with water velocity, grain size scores, substrate richness, or edge-interior scores within central Ohio riffles in contrast to our observed relationships of *C. cornutus* hellgrammite occurrence and density with these environmental factors during the early summer. Perhaps once *C. cornutus* hellgrammites select a plot based on water velocity and grain size scores, their subsequent growth is a function of other unmeasured variables, such as food availability or water temperature. This conjecture is supported by Corydalinae hellgrammites’ preference for a stationary existence and their aversion to relocating to different locations within a riffle even when faced with starvation [15]. However, information on Corydalinae hellgrammite movement is very limited and derives from one short-term study in Japan [15], and subsequently there are notable information gaps on when and where larval Corydalinae hellgrammite movement occurs within streams. Life history information suggests that final instar Corydalinae hellgrammites will move toward the riffle’s edge to prepare for pupation [3,32]. Details related to the timing and extent of hellgrammite movement need to be addressed by future research.

In conclusion, our results documented the importance of water velocity and grain size score on *C. cornutus* hellgrammite occurrence and density within 1-m^2^ plots in central Ohio riffles during the early summer. Additionally, specific *C. cornutus* habitat relationships observed included: (1) increases in occurrence were observed with increasing water velocity, grain size score, and substrate richness; (2) increases in density occurred with increases in the same three variables as well as edge-interior scores; and (3) increases in body size occurred with increasing distance to the nearest plot with hellgrammites. These findings provide new insights regarding *C. cornutus* hellgrammite habitat relationships within riffles in the northern part of this species’ range in the United States during the early summer. Our results highlighting the importance of water velocity and grain size scores suggest that *C. cornutus* hellgrammites can function as an indicator species that will reflect the water velocity and substrate conditions within riffles in the Midwestern United States. Our results also suggest that stream habitat management efforts that increase water velocity and grain size scores within degraded riffles will benefit *C. cornutus* hellgrammite populations in Midwestern U.S. rivers.

## Figures and Tables

**Table 1:** Summary of selected watershed and hydrologic characteristics of riffles selected as study sites in Alum Creek (AC1, AC2), Upper Big Walnut Creek (BW1, BW2), Kokosing River (KR1, KR2, KR3), and Clear Fork River (CF1, CF2, CF3), Ohio, 2023–2024. Stream order [25] and watershed size were obtained from Stroud Water Research Center [26] and percent land use data were acquired from USDA NASS [27]. Hydrologic variables reported are means of measurements obtained from June to July 2023 and 2024 with exception of site KR2 that was only sampled in 2024.

	AC1	AC2	BW1	BW2	KR1	KR2	KR3	CF1	CF2	CF3
Strahler stream order	4	4	4	4	4	5	5	4	4	4
Watershed size (km^2^)	67	65	282	282	192	678	1226	515	518	528
Percent forest	30.91	31.33	26.75	26.75	34.55	30.53	32.06	44.78	44.82	45.20
Percent agriculture	59.53	58.88	64.35	64.33	54.58	59.11	58.12	42.58	42.55	42.23
Percent urban	9.10	9.32	8.51	8.53	9.54	9.29	8.82	10.73	10.73	10.67
Mean riffle length (m)	18.30	28.35	32.32	31.68	22.62	31.93	58.54	26.80	70.29	34.50
Mean wetted width (m)	12.63	12.36	10.55	10.64	11.51	20.23	40.31	21.13	19.58	19.31
Mean discharge (m^3^/s)	0.43	0.33	0.33	0.42	0.75	1.85	4.68	2.04	2.38	2.64

**Table 2:** Fixed effects coefficients and *p* values from mixed effects model analyses to evaluate the relationships of *C. cornutus* hellgrammite occurrence, density, and head capsule width with selected habitat variables in riffles within Alum Creek, Upper Big Walnut Creek, Kokosing River, and Clear Fork River, Ohio, 2023–2024. Bolded coefficients and *p* values signify those fixed effects with *p* < 0.05.

Response Variable	Fixed Effect	Coefficient	*p* Value
Occurrence	Water velocity	**3.274**	**<0.001**
	Grain size score	**0.008**	**0.001**
	Substrate richness	**0.540**	**0.012**
	Edge-interior score	1.892	0.053
Density	Water velocity	**1.212**	**<0.001**
	Grain size score	**0.004**	**0.001**
	Substrate richness	**0.244**	**0.025**
	Edge-interior score	**0.978**	**0.038**
Head capsule width	Water velocity	0.022	0.666
	Grain size score	0.000	0.502
	Distance to nearest plot with hellgrammites	**0.007**	**0.007**

## Data Availability

The original contributions presented in this study are included in the article/Supplementary Materials. Further inquiries can be directed to the corresponding author.

## Supplementary Materials

The following supporting information can be downloaded at: https://www.mdpi.com/article/10.3390/insects17040410/s1, Supplemental File S1: Raw data used in the statistical analyses; Supplemental File S2: R code used as part of the statistical analyses.

## Abbreviations

The following abbreviations are used in this manuscript:

OH	Ohio
USA	United States of America
USDA	United States Department of Agriculture
U.S.	United States
NASS	National Agricultural Statistics Service
AIC	Akaike’s Information Criterion
Wvel	Water velocity
GSS	Grain size score
