# Supplementary material for: Small-Scale Habitat Relationships of Corydalus cornutus Hellgrammites in Central Ohio Riffles
Source: Insects. 2026 Apr 10;17(4):410. doi: 10.3390/insects17040410 (PMC13115823; doi:10.3390/insects17040410)
Supplement: Supplementary file 1 [file insects-17-00410-s001.zip › insects-4189354-supplementary/BossleyetalSupplementaryFile1-Insects.pdf]

Supplementary File S1. Data associated with Bossley et al. 2026. Small-scale habitat relationships of *Corydalus cornutus* hellgrammites in central Ohio riffles. *Insects.*

Part 1 - *Corydalus cornutus* hellgrammite response variables												
SiteN	WS	Date	Year	Ycat	Transect	Plot	pDen	pmnBL	pmnHCW	lgHCW	pOCR	
KR1	KOKO1	6/5/2023	2023	2023A	DT	P1	0	NA	NA	NA	0	
KR1	KOKO1	6/5/2023	2023	2023A	DT	P2	1	65.09	6.35	0.866	1	
KR1	KOKO1	6/5/2023	2023	2023A	DT	P3	0	NA	NA	NA	0	
KR1	KOKO1	6/5/2023	2023	2023A	DT	P4	0	NA	NA	NA	0	
KR1	KOKO1	6/5/2023	2023	2023A	DT	P5	0	NA	NA	NA	0	
KR1	KOKO1	6/5/2023	2023	2023A	MT	P1	0	NA	NA	NA	0	
KR1	KOKO1	6/5/2023	2023	2023A	MT	P2	0	NA	NA	NA	0	
KR1	KOKO1	6/5/2023	2023	2023A	MT	P3	0	NA	NA	NA	0	
KR1	KOKO1	6/5/2023	2023	2023A	MT	P4	0	NA	NA	NA	0	
KR1	KOKO1	6/5/2023	2023	2023A	MT	P5	1	30.16	3.18	0.621	1	
KR1	KOKO1	6/5/2023	2023	2023A	UT	P1	0	NA	NA	NA	0	
KR1	KOKO1	6/5/2023	2023	2023A	UT	P2	0	NA	NA	NA	0	
KR1	KOKO1	6/5/2023	2023	2023A	UT	P3	0	NA	NA	NA	0	
KR1	KOKO1	6/5/2023	2023	2023A	UT	P4	0	NA	NA	NA	0	
KR1	KOKO1	6/5/2023	2023	2023A	UT	P5	0	NA	NA	NA	0	
KR1	KOKO1	6/5/2023	2023	2023A	SS	SS1	0	NA	NA	NA	0	
KR1	KOKO1	6/5/2023	2023	2023A	SS	SS2	0	NA	NA	NA	0	
KR1	KOKO1	6/5/2023	2023	2023A	SS	SS3	0	NA	NA	NA	0	
KR1	KOKO1	6/5/2023	2023	2023A	SS	SS4	0	NA	NA	NA	0	
KR1	KOKO1	6/5/2023	2023	2023A	SS	SS5	0	NA	NA	NA	0	
KR3	KOKO1	6/6/2023	2023	2023A	DT	P1	0	NA	NA	NA	0	
KR3	KOKO1	6/6/2023	2023	2023A	DT	P2	0	NA	NA	NA	0	
KR3	KOKO1	6/6/2023	2023	2023A	DT	P3	0	NA	NA	NA	0	
KR3	KOKO1	6/6/2023	2023	2023A	DT	P4	0	NA	NA	NA	0	
KR3	KOKO1	6/6/2023	2023	2023A	DT	P5	0	NA	NA	NA	0	
KR3	KOKO1	6/6/2023	2023	2023A	MT	P1	0	NA	NA	NA	0	
KR3	KOKO1	6/6/2023	2023	2023A	MT	P2	0	NA	NA	NA	0	

SiteN	WS	Date	Year	Ycat	Transect	Plot	pDen	pmnBL	pmnHCW	lgHCW	pOCR	
KR3	KOKO1	6/6/2023	2023	2023A	MT	P3	2	42.5	3.5	0.653	1	
KR3	KOKO1	6/6/2023	2023	2023A	MT	P4	5	52.4	5.4	0.806	1	
KR3	KOKO1	6/6/2023	2023	2023A	MT	P5	0	NA	NA	NA	0	
KR3	KOKO1	6/6/2023	2023	2023A	UT	P1	0	NA	NA	NA	0	
KR3	KOKO1	6/6/2023	2023	2023A	UT	P2	0	NA	NA	NA	0	
KR3	KOKO1	6/6/2023	2023	2023A	UT	P3	0	NA	NA	NA	0	
KR3	KOKO1	6/6/2023	2023	2023A	UT	P4	0	NA	NA	NA	0	
KR3	KOKO1	6/6/2023	2023	2023A	UT	P5	0	NA	NA	NA	0	
KR3	KOKO1	6/6/2023	2023	2023A	SS	SS1	2	45	3.5	0.653	1	
KR3	KOKO1	6/6/2023	2023	2023A	SS	SS2	0	NA	NA	NA	0	
KR3	KOKO1	6/6/2023	2023	2023A	SS	SS3	0	NA	NA	NA	0	
KR3	KOKO1	6/6/2023	2023	2023A	SS	SS4	1	50	6	0.845	1	
KR3	KOKO1	6/6/2023	2023	2023A	SS	SS5	5	60	6.3	0.863	1	
AC1	ALUM1	6/29/2023	2023	2023A	DT	P1	0	NA	NA	NA	0	
AC1	ALUM1	6/29/2023	2023	2023A	DT	P2	0	NA	NA	NA	0	
AC1	ALUM1	6/29/2023	2023	2023A	DT	P3	0	NA	NA	NA	0	
AC1	ALUM1	6/29/2023	2023	2023A	DT	P4	0	NA	NA	NA	0	
AC1	ALUM1	6/29/2023	2023	2023A	DT	P5	0	NA	NA	NA	0	
AC1	ALUM1	6/29/2023	2023	2023A	MT	P1	0	NA	NA	NA	0	
AC1	ALUM1	6/29/2023	2023	2023A	MT	P2	0	NA	NA	NA	0	
AC1	ALUM1	6/29/2023	2023	2023A	MT	P3	0	NA	NA	NA	0	
AC1	ALUM1	6/29/2023	2023	2023A	MT	P4	1	40	4	0.699	1	
AC1	ALUM1	6/29/2023	2023	2023A	MT	P5	0	NA	NA	NA	0	
AC1	ALUM1	6/29/2023	2023	2023A	UT	P1	0	NA	NA	NA	0	
AC1	ALUM1	6/29/2023	2023	2023A	UT	P2	0	NA	NA	NA	0	
AC1	ALUM1	6/29/2023	2023	2023A	UT	P3	0	NA	NA	NA	0	
AC1	ALUM1	6/29/2023	2023	2023A	UT	P4	0	NA	NA	NA	0	
AC1	ALUM1	6/29/2023	2023	2023A	UT	P5	0	NA	NA	NA	0	
AC1	ALUM1	6/29/2023	2023	2023A	SS	SS1	0	NA	NA	NA	0	
AC1	ALUM1	6/29/2023	2023	2023A	SS	SS2	0	NA	NA	NA	0	
AC1	ALUM1	6/29/2023	2023	2023A	SS	SS3	0	NA	NA	NA	0	

SiteN	WS	Date	Year	Ycat	Transect	Plot	pDen	pmnBL	pmnHCW	lgHCW	pOCR	
AC1	ALUM1	6/29/2023	2023	2023A	SS	SS4	1	39	3	0.602	1	
AC1	ALUM1	6/29/2023	2023	2023A	SS	SS5	0	NA	NA	NA	0	
AC2	ALUM1	6/29/2023	2023	2023A	DT	P1	0	NA	NA	NA	0	
AC2	ALUM1	6/29/2023	2023	2023A	DT	P2	0	NA	NA	NA	0	
AC2	ALUM1	6/29/2023	2023	2023A	DT	P3	0	NA	NA	NA	0	
AC2	ALUM1	6/29/2023	2023	2023A	DT	P4	0	NA	NA	NA	0	
AC2	ALUM1	6/29/2023	2023	2023A	DT	P5	0	NA	NA	NA	0	
AC2	ALUM1	6/29/2023	2023	2023A	MT	P1	0	NA	NA	NA	0	
AC2	ALUM1	6/29/2023	2023	2023A	MT	P2	0	NA	NA	NA	0	
AC2	ALUM1	6/29/2023	2023	2023A	MT	P3	0	NA	NA	NA	0	
AC2	ALUM1	6/29/2023	2023	2023A	MT	P4	1	35	3	0.602	1	
AC2	ALUM1	6/29/2023	2023	2023A	MT	P5	0	NA	NA	NA	0	
AC2	ALUM1	6/29/2023	2023	2023A	UT	P1	0	NA	NA	NA	0	
AC2	ALUM1	6/29/2023	2023	2023A	UT	P2	0	NA	NA	NA	0	
AC2	ALUM1	6/29/2023	2023	2023A	UT	P3	0	NA	NA	NA	0	
AC2	ALUM1	6/29/2023	2023	2023A	UT	P4	0	NA	NA	NA	0	
AC2	ALUM1	6/29/2023	2023	2023A	UT	P5	0	NA	NA	NA	0	
AC2	ALUM1	6/29/2023	2023	2023A	SS	SS1	0	NA	NA	NA	0	
AC2	ALUM1	6/29/2023	2023	2023A	SS	SS2	1	20.5	3	0.602	1	
AC2	ALUM1	6/29/2023	2023	2023A	SS	SS3	0	NA	NA	NA	0	
AC2	ALUM1	6/29/2023	2023	2023A	SS	SS4	0	NA	NA	NA	0	
AC2	ALUM1	6/29/2023	2023	2023A	SS	SS5	0	NA	NA	NA	0	
CF3	MOHI1	7/7/2023	2023	2023A	DT	P1	10	42.9	4.8	0.763	1	
CF3	MOHI1	7/7/2023	2023	2023A	DT	P2	4	23.5	2.38	0.529	1	
CF3	MOHI1	7/7/2023	2023	2023A	DT	P3	2	54.5	7.75	0.942	1	
CF3	MOHI1	7/7/2023	2023	2023A	DT	P4	0	NA	NA	NA	0	
CF3	MOHI1	7/7/2023	2023	2023A	DT	P5	0	NA	NA	NA	0	
CF3	MOHI1	7/7/2023	2023	2023A	MT	P1	2	74.5	9	1	1	
CF3	MOHI1	7/7/2023	2023	2023A	MT	P2	2	26	2.5	0.544	1	
CF3	MOHI1	7/7/2023	2023	2023A	MT	P3	3	26.33	2.67	0.565	1	
CF3	MOHI1	7/7/2023	2023	2023A	MT	P4	1	37	4.5	0.74	1	

SiteN	WS	Date	Year	Ycat	Transect	Plot	pDen	pmnBL	pmnHCW	lgHCW	pOCR	
CF3	MOHI1	7/7/2023	2023	2023A	MT	P5	0	NA	NA	NA	0	
CF3	MOHI1	7/7/2023	2023	2023A	UT	P1	1	61	7	0.903	1	
CF3	MOHI1	7/7/2023	2023	2023A	UT	P2	2	42	6.25	0.86	1	
CF3	MOHI1	7/7/2023	2023	2023A	UT	P3	3	45	4.67	0.754	1	
CF3	MOHI1	7/7/2023	2023	2023A	UT	P4	1	68	8	0.954	1	
CF3	MOHI1	7/7/2023	2023	2023A	UT	P5	1	35	3	0.602	1	
CF3	MOHI1	7/7/2023	2023	2023A	SS	SS1	1	40	4.5	0.74	1	
CF3	MOHI1	7/7/2023	2023	2023A	SS	SS2	0	NA	NA	NA	0	
CF3	MOHI1	7/7/2023	2023	2023A	SS	SS3	2	30.5	3.5	0.653	1	
CF3	MOHI1	7/7/2023	2023	2023A	SS	SS4	1	45	4.5	0.74	1	
CF3	MOHI1	7/7/2023	2023	2023A	SS	SS5	1	64	9.5	1.021	1	
CF2	MOHI1	7/8/2023	2023	2023A	DT	P1	0	NA	NA	NA	0	
CF2	MOHI1	7/8/2023	2023	2023A	DT	P2	2	48	6.5	0.875	1	
CF2	MOHI1	7/8/2023	2023	2023A	DT	P3	0	NA	NA	NA	0	
CF2	MOHI1	7/8/2023	2023	2023A	DT	P4	0	NA	NA	NA	0	
CF2	MOHI1	7/8/2023	2023	2023A	DT	P5	0	NA	NA	NA	0	
CF2	MOHI1	7/8/2023	2023	2023A	MT	P1	1	43	5	0.778	1	
CF2	MOHI1	7/8/2023	2023	2023A	MT	P2	0	NA	NA	NA	0	
CF2	MOHI1	7/8/2023	2023	2023A	MT	P3	1	40	3.5	0.653	1	
CF2	MOHI1	7/8/2023	2023	2023A	MT	P4	2	44	3.75	0.677	1	
CF2	MOHI1	7/8/2023	2023	2023A	MT	P5	0	NA	NA	NA	0	
CF2	MOHI1	7/8/2023	2023	2023A	UT	P1	0	NA	NA	NA	0	
CF2	MOHI1	7/8/2023	2023	2023A	UT	P2	1	21	2	0.477	1	
CF2	MOHI1	7/8/2023	2023	2023A	UT	P3	0	NA	NA	NA	0	
CF2	MOHI1	7/8/2023	2023	2023A	UT	P4	1	50	5	0.778	1	
CF2	MOHI1	7/8/2023	2023	2023A	UT	P5	0	NA	NA	NA	0	
CF2	MOHI1	7/8/2023	2023	2023A	SS	SS1	5	46	4.1	0.708	1	
CF2	MOHI1	7/8/2023	2023	2023A	SS	SS2	2	56.5	6.25	0.86	1	
CF2	MOHI1	7/8/2023	2023	2023A	SS	SS3	1	49	4.5	0.74	1	
CF2	MOHI1	7/8/2023	2023	2023A	SS	SS4	2	60.5	7.5	0.929	1	
CF2	MOHI1	7/8/2023	2023	2023A	SS	SS5	0	NA	NA	NA	0	

SiteN	WS	Date	Year	Ycat	Transect	Plot	pDen	pmnBL	pmnHCW	lgHCW	pOCR	
BW2	UBWC1	7/11/2023	2023	2023A	DT	P1	0	NA	NA	NA	0	
BW2	UBWC1	7/11/2023	2023	2023A	DT	P2	0	NA	NA	NA	0	
BW2	UBWC1	7/11/2023	2023	2023A	DT	P3	0	NA	NA	NA	0	
BW2	UBWC1	7/11/2023	2023	2023A	DT	P4	0	NA	NA	NA	0	
BW2	UBWC1	7/11/2023	2023	2023A	DT	P5	0	NA	NA	NA	0	
BW2	UBWC1	7/11/2023	2023	2023A	MT	P1	0	NA	NA	NA	0	
BW2	UBWC1	7/11/2023	2023	2023A	MT	P2	0	NA	NA	NA	0	
BW2	UBWC1	7/11/2023	2023	2023A	MT	P3	0	NA	NA	NA	0	
BW2	UBWC1	7/11/2023	2023	2023A	MT	P4	0	NA	NA	NA	0	
BW2	UBWC1	7/11/2023	2023	2023A	MT	P5	0	NA	NA	NA	0	
BW2	UBWC1	7/11/2023	2023	2023A	UT	P1	0	NA	NA	NA	0	
BW2	UBWC1	7/11/2023	2023	2023A	UT	P2	0	NA	NA	NA	0	
BW2	UBWC1	7/11/2023	2023	2023A	UT	P3	0	NA	NA	NA	0	
BW2	UBWC1	7/11/2023	2023	2023A	UT	P4	0	NA	NA	NA	0	
BW2	UBWC1	7/11/2023	2023	2023A	UT	P5	0	NA	NA	NA	0	
BW2	UBWC1	7/11/2023	2023	2023A	SS	SS1	0	NA	NA	NA	0	
BW2	UBWC1	7/11/2023	2023	2023A	SS	SS2	0	NA	NA	NA	0	
BW2	UBWC1	7/11/2023	2023	2023A	SS	SS3	0	NA	NA	NA	0	
BW2	UBWC1	7/11/2023	2023	2023A	SS	SS4	0	NA	NA	NA	0	
BW2	UBWC1	7/11/2023	2023	2023A	SS	SS5	0	NA	NA	NA	0	
BW1	UBWC1	7/11/2023	2023	2023A	DT	P1	0	NA	NA	NA	0	
BW1	UBWC1	7/11/2023	2023	2023A	DT	P2	0	NA	NA	NA	0	
BW1	UBWC1	7/11/2023	2023	2023A	DT	P3	0	NA	NA	NA	0	
BW1	UBWC1	7/11/2023	2023	2023A	DT	P4	0	NA	NA	NA	0	
BW1	UBWC1	7/11/2023	2023	2023A	DT	P5	0	NA	NA	NA	0	
BW1	UBWC1	7/11/2023	2023	2023A	MT	P1	0	NA	NA	NA	0	
BW1	UBWC1	7/11/2023	2023	2023A	MT	P2	0	NA	NA	NA	0	
BW1	UBWC1	7/11/2023	2023	2023A	MT	P3	0	NA	NA	NA	0	
BW1	UBWC1	7/11/2023	2023	2023A	MT	P4	0	NA	NA	NA	0	
BW1	UBWC1	7/11/2023	2023	2023A	MT	P5	0	NA	NA	NA	0	
BW1	UBWC1	7/11/2023	2023	2023A	UT	P1	0	NA	NA	NA	0	

SiteN	WS	Date	Year	Ycat	Transect	Plot	pDen	pmnBL	pmnHCW	lgHCW	pOCR	
BW1	UBWC1	7/11/2023	2023	2023A	UT	P2	0	NA	NA	NA	0	
BW1	UBWC1	7/11/2023	2023	2023A	UT	P3	0	NA	NA	NA	0	
BW1	UBWC1	7/11/2023	2023	2023A	UT	P4	0	NA	NA	NA	0	
BW1	UBWC1	7/11/2023	2023	2023A	UT	P5	0	NA	NA	NA	0	
BW1	UBWC1	7/11/2023	2023	2023A	SS	SS1	0	NA	NA	NA	0	
BW1	UBWC1	7/11/2023	2023	2023A	SS	SS2	0	NA	NA	NA	0	
BW1	UBWC1	7/11/2023	2023	2023A	SS	SS3	0	NA	NA	NA	0	
BW1	UBWC1	7/11/2023	2023	2023A	SS	SS4	0	NA	NA	NA	0	
BW1	UBWC1	7/11/2023	2023	2023A	SS	SS5	0	NA	NA	NA	0	
CF1	MOHI1	7/19/2023	2023	2023A	DT	P1	0	NA	NA	NA	0	
CF1	MOHI1	7/19/2023	2023	2023A	DT	P2	0	NA	NA	NA	0	
CF1	MOHI1	7/19/2023	2023	2023A	DT	P3	2	41.5	6.25	0.86	1	
CF1	MOHI1	7/19/2023	2023	2023A	DT	P4	1	42	4.5	0.74	1	
CF1	MOHI1	7/19/2023	2023	2023A	DT	P5	1	52	6	0.845	1	
CF1	MOHI1	7/19/2023	2023	2023A	MT	P1	1	30	3.03	0.605	1	
CF1	MOHI1	7/19/2023	2023	2023A	MT	P2	4	52.75	7.25	0.916	1	
CF1	MOHI1	7/19/2023	2023	2023A	MT	P3	3	47	5.33	0.801	1	
CF1	MOHI1	7/19/2023	2023	2023A	MT	P4	2	48	5.5	0.813	1	
CF1	MOHI1	7/19/2023	2023	2023A	MT	P5	0	NA	NA	NA	0	
CF1	MOHI1	7/19/2023	2023	2023A	UT	P1	2	63.5	7.25	0.916	1	
CF1	MOHI1	7/19/2023	2023	2023A	UT	P2	4	32.67	3.83	0.684	1	
CF1	MOHI1	7/19/2023	2023	2023A	UT	P3	1	61	7.5	0.929	1	
CF1	MOHI1	7/19/2023	2023	2023A	UT	P4	2	32	3.25	0.628	1	
CF1	MOHI1	7/19/2023	2023	2023A	UT	P5	0	NA	NA	NA	0	
CF1	MOHI1	7/19/2023	2023	2023A	SS	SS1	3	57	6	0.845	1	
CF1	MOHI1	7/19/2023	2023	2023A	SS	SS2	4	37.75	4.5	0.74	1	
CF1	MOHI1	7/19/2023	2023	2023A	SS	SS3	11	42.14	4.98	0.777	1	
CF1	MOHI1	7/19/2023	2023	2023A	SS	SS4	7	44.29	4.79	0.763	1	
CF1	MOHI1	7/19/2023	2023	2023A	SS	SS5	8	47.13	5.75	0.829	1	
KR1	KOKO1	6/4/2024	2024	2024B	DT	P1	0	NA	NA	NA	0	
KR1	KOKO1	6/4/2024	2024	2024B	DT	P2	0	NA	NA	NA	0	

SiteN	WS	Date	Year	Ycat	Transect	Plot	pDen	pmnBL	pmnHCW	lgHCW	pOCR	
KR1	KOKO1	6/4/2024	2024	2024B	DT	P3	0	NA	NA	NA	0	
KR1	KOKO1	6/4/2024	2024	2024B	DT	P4	0	NA	NA	NA	0	
KR1	KOKO1	6/4/2024	2024	2024B	DT	P5	0	NA	NA	NA	0	
KR1	KOKO1	6/4/2024	2024	2024B	MT	P6	0	NA	NA	NA	0	
KR1	KOKO1	6/4/2024	2024	2024B	MT	P7	0	NA	NA	NA	0	
KR1	KOKO1	6/4/2024	2024	2024B	MT	P8	0	NA	NA	NA	0	
KR1	KOKO1	6/4/2024	2024	2024B	MT	P9	0	NA	NA	NA	0	
KR1	KOKO1	6/4/2024	2024	2024B	MT	P10	0	NA	NA	NA	0	
KR1	KOKO1	6/4/2024	2024	2024B	UT	P11	0	NA	NA	NA	0	
KR1	KOKO1	6/4/2024	2024	2024B	UT	P12	0	NA	NA	NA	0	
KR1	KOKO1	6/4/2024	2024	2024B	UT	P13	0	NA	NA	NA	0	
KR1	KOKO1	6/4/2024	2024	2024B	UT	P14	0	NA	NA	NA	0	
KR1	KOKO1	6/4/2024	2024	2024B	UT	P15	0	NA	NA	NA	0	
KR1	KOKO1	6/4/2024	2024	2024B	SS	P16	0	NA	NA	NA	0	
KR1	KOKO1	6/4/2024	2024	2024B	SS	P17	0	NA	NA	NA	0	
KR1	KOKO1	6/4/2024	2024	2024B	SS	P18	0	NA	NA	NA	0	
KR1	KOKO1	6/4/2024	2024	2024B	SS	P19	1	90	10	1.041	1	
KR1	KOKO1	6/4/2024	2024	2024B	SS	P20	1	55	5	0.778	1	
KR3	KOKO1	6/25/2024	2024	2024B	DT	P1	0	NA	NA	NA	0	
KR3	KOKO1	6/25/2024	2024	2024B	DT	P2	0	NA	NA	NA	0	
KR3	KOKO1	6/25/2024	2024	2024B	DT	P3	0	NA	NA	NA	0	
KR3	KOKO1	6/25/2024	2024	2024B	DT	P4	0	NA	NA	NA	0	
KR3	KOKO1	6/25/2024	2024	2024B	DT	P5	0	NA	NA	NA	0	
KR3	KOKO1	6/25/2024	2024	2024B	MT	P6	0	NA	NA	NA	0	
KR3	KOKO1	6/25/2024	2024	2024B	MT	P7	0	NA	NA	NA	0	
KR3	KOKO1	6/25/2024	2024	2024B	MT	P8	0	NA	NA	NA	0	
KR3	KOKO1	6/25/2024	2024	2024B	MT	P9	0	NA	NA	NA	0	
KR3	KOKO1	6/25/2024	2024	2024B	MT	P10	0	NA	NA	NA	0	
KR3	KOKO1	6/25/2024	2024	2024B	UT	P11	0	NA	NA	NA	0	
KR3	KOKO1	6/25/2024	2024	2024B	UT	P12	0	NA	NA	NA	0	
KR3	KOKO1	6/25/2024	2024	2024B	UT	P13	0	NA	NA	NA	0	

SiteN	WS	Date	Year	Ycat	Transect	Plot	pDen	pmnBL	pmnHCW	lgHCW	pOCR	
KR3	KOKO1	6/25/2024	2024	2024B	UT	P14	0	NA	NA	NA	0	
KR3	KOKO1	6/25/2024	2024	2024B	UT	P15	0	NA	NA	NA	0	
KR3	KOKO1	6/25/2024	2024	2024B	SS	P16	2	64	8.25	0.966	1	
KR3	KOKO1	6/25/2024	2024	2024B	SS	P17	0	NA	NA	NA	0	
KR3	KOKO1	6/25/2024	2024	2024B	SS	P18	0	NA	NA	NA	0	
KR3	KOKO1	6/25/2024	2024	2024B	SS	P19	1	65	6	0.845	1	
KR3	KOKO1	6/25/2024	2024	2024B	SS	P20	2	70	9	1	1	
AC1	ALUM1	6/10/2024	2024	2024B	DT	P1	0	NA	NA	NA	0	
AC1	ALUM1	6/10/2024	2024	2024B	DT	P2	0	NA	NA	NA	0	
AC1	ALUM1	6/10/2024	2024	2024B	DT	P3	0	NA	NA	NA	0	
AC1	ALUM1	6/10/2024	2024	2024B	DT	P4	0	NA	NA	NA	0	
AC1	ALUM1	6/10/2024	2024	2024B	DT	P5	0	NA	NA	NA	0	
AC1	ALUM1	6/10/2024	2024	2024B	MT	P6	0	NA	NA	NA	0	
AC1	ALUM1	6/10/2024	2024	2024B	MT	P7	0	NA	NA	NA	0	
AC1	ALUM1	6/10/2024	2024	2024B	MT	P8	0	NA	NA	NA	0	
AC1	ALUM1	6/10/2024	2024	2024B	MT	P9	1	30	3	0.602	1	
AC1	ALUM1	6/10/2024	2024	2024B	MT	P10	0	NA	NA	NA	0	
AC1	ALUM1	6/10/2024	2024	2024B	UT	P11	0	NA	NA	NA	0	
AC1	ALUM1	6/10/2024	2024	2024B	UT	P12	0	NA	NA	NA	0	
AC1	ALUM1	6/10/2024	2024	2024B	UT	P13	0	NA	NA	NA	0	
AC1	ALUM1	6/10/2024	2024	2024B	UT	P14	0	NA	NA	NA	0	
AC1	ALUM1	6/10/2024	2024	2024B	UT	P15	0	NA	NA	NA	0	
AC1	ALUM1	6/10/2024	2024	2024B	SS	P16	0	NA	NA	NA	0	
AC1	ALUM1	6/10/2024	2024	2024B	SS	P17	0	NA	NA	NA	0	
AC1	ALUM1	6/10/2024	2024	2024B	SS	P18	0	NA	NA	NA	0	
AC1	ALUM1	6/10/2024	2024	2024B	SS	P19	0	NA	NA	NA	0	
AC1	ALUM1	6/10/2024	2024	2024B	SS	P20	0	NA	NA	NA	0	
AC2	ALUM1	6/11/2024	2024	2024B	DT	P1	0	NA	NA	NA	0	
AC2	ALUM1	6/11/2024	2024	2024B	DT	P2	0	NA	NA	NA	0	
AC2	ALUM1	6/11/2024	2024	2024B	DT	P3	0	NA	NA	NA	0	
AC2	ALUM1	6/11/2024	2024	2024B	DT	P4	0	NA	NA	NA	0	

SiteN	WS	Date	Year	Ycat	Transect	Plot	pDen	pmnBL	pmnHCW	lgHCW	pOCR	
AC2	ALUM1	6/11/2024	2024	2024B	DT	P5	0	NA	NA	NA	0	
AC2	ALUM1	6/11/2024	2024	2024B	MT	P6	0	NA	NA	NA	0	
AC2	ALUM1	6/11/2024	2024	2024B	MT	P7	0	NA	NA	NA	0	
AC2	ALUM1	6/11/2024	2024	2024B	MT	P8	0	NA	NA	NA	0	
AC2	ALUM1	6/11/2024	2024	2024B	MT	P9	0	NA	NA	NA	0	
AC2	ALUM1	6/11/2024	2024	2024B	MT	P10	0	NA	NA	NA	0	
AC2	ALUM1	6/11/2024	2024	2024B	UT	P11	0	NA	NA	NA	0	
AC2	ALUM1	6/11/2024	2024	2024B	UT	P12	0	NA	NA	NA	0	
AC2	ALUM1	6/11/2024	2024	2024B	UT	P13	0	NA	NA	NA	0	
AC2	ALUM1	6/11/2024	2024	2024B	UT	P14	0	NA	NA	NA	0	
AC2	ALUM1	6/11/2024	2024	2024B	UT	P15	0	NA	NA	NA	0	
AC2	ALUM1	6/11/2024	2024	2024B	SS	P16	0	NA	NA	NA	0	
AC2	ALUM1	6/11/2024	2024	2024B	SS	P17	0	NA	NA	NA	0	
AC2	ALUM1	6/11/2024	2024	2024B	SS	P18	0	NA	NA	NA	0	
AC2	ALUM1	6/11/2024	2024	2024B	SS	P19	0	NA	NA	NA	0	
AC2	ALUM1	6/11/2024	2024	2024B	SS	P20	0	NA	NA	NA	0	
CF3	MOHI1	6/17/2024	2024	2024B	DT	P1	2	57.5	6.5	0.875	1	
CF3	MOHI1	6/17/2024	2024	2024B	DT	P2	10	35.5	3.63	0.666	1	
CF3	MOHI1	6/17/2024	2024	2024B	DT	P3	4	27	2.67	0.565	1	
CF3	MOHI1	6/17/2024	2024	2024B	DT	P4	0	NA	NA	NA	0	
CF3	MOHI1	6/17/2024	2024	2024B	DT	P5	0	NA	NA	NA	0	
CF3	MOHI1	6/17/2024	2024	2024B	MT	P6	4	50.67	5	0.778	1	
CF3	MOHI1	6/17/2024	2024	2024B	MT	P7	1	18	1.5	0.398	1	
CF3	MOHI1	6/17/2024	2024	2024B	MT	P8	5	38	4.5	0.74	1	
CF3	MOHI1	6/17/2024	2024	2024B	MT	P9	1	49	7	0.903	1	
CF3	MOHI1	6/17/2024	2024	2024B	MT	P10	0	NA	NA	NA	0	
CF3	MOHI1	6/17/2024	2024	2024B	UT	P11	4	37	4.13	0.71	1	
CF3	MOHI1	6/17/2024	2024	2024B	UT	P12	3	19.67	1.83	0.452	1	
CF3	MOHI1	6/17/2024	2024	2024B	UT	P13	5	42.6	5.2	0.792	1	
CF3	MOHI1	6/17/2024	2024	2024B	UT	P14	1	34	3	0.602	1	
CF3	MOHI1	6/17/2024	2024	2024B	UT	P15	0	NA	NA	NA	0	

SiteN	WS	Date	Year	Ycat	Transect	Plot	pDen	pmnBL	pmnHCW	lgHCW	pOCR	
CF3	MOHI1	6/17/2024	2024	2024B	SS	P16	5	41.8	5.1	0.785	1	
CF3	MOHI1	6/17/2024	2024	2024B	SS	P17	6	31.83	3.25	0.628	1	
CF3	MOHI1	6/17/2024	2024	2024B	SS	P18	5	31	3.4	0.643	1	
CF3	MOHI1	6/17/2024	2024	2024B	SS	P19	1	20	2	0.477	1	
CF3	MOHI1	6/17/2024	2024	2024B	SS	P20	3	35	3.17	0.62	1	
CF2	MOHI1	7/1/2024	2024	2024B	DT	P1	1	26	2.5	0.544	1	
CF2	MOHI1	7/1/2024	2024	2024B	DT	P2	4	48	5.13	0.787	1	
CF2	MOHI1	7/1/2024	2024	2024B	DT	P3	5	29.4	3	0.602	1	
CF2	MOHI1	7/1/2024	2024	2024B	DT	P4	0	NA	NA	NA	0	
CF2	MOHI1	7/1/2024	2024	2024B	DT	P5	0	NA	NA	NA	0	
CF2	MOHI1	7/1/2024	2024	2024B	MT	P6	1	NA	NA	NA	1	
CF2	MOHI1	7/1/2024	2024	2024B	MT	P7	0	NA	NA	NA	0	
CF2	MOHI1	7/1/2024	2024	2024B	MT	P8	3	40	4	0.699	1	
CF2	MOHI1	7/1/2024	2024	2024B	MT	P9	2	39.5	3.5	0.653	1	
CF2	MOHI1	7/1/2024	2024	2024B	MT	P10	0	NA	NA	NA	0	
CF2	MOHI1	7/1/2024	2024	2024B	UT	P11	0	NA	NA	NA	0	
CF2	MOHI1	7/1/2024	2024	2024B	UT	P12	1	33	3.5	0.653	1	
CF2	MOHI1	7/1/2024	2024	2024B	UT	P13	1	35	3.5	0.653	1	
CF2	MOHI1	7/1/2024	2024	2024B	UT	P14	0	NA	NA	NA	0	
CF2	MOHI1	7/1/2024	2024	2024B	UT	P15	1	36	3.5	0.653	1	
CF2	MOHI1	7/1/2024	2024	2024B	SS	P16	5	40.4	4.5	0.74	1	
CF2	MOHI1	7/1/2024	2024	2024B	SS	P17	2	23.5	2.25	0.512	1	
CF2	MOHI1	7/1/2024	2024	2024B	SS	P18	7	58.86	6.57	0.879	1	
CF2	MOHI1	7/1/2024	2024	2024B	SS	P19	1	80	9	1	1	
CF2	MOHI1	7/1/2024	2024	2024B	SS	P20	0	NA	NA	NA	0	
BW2	UBWC1	6/18/2024	2024	2024B	DT	P1	0	NA	NA	NA	0	
BW2	UBWC1	6/18/2024	2024	2024B	DT	P2	0	NA	NA	NA	0	
BW2	UBWC1	6/18/2024	2024	2024B	DT	P3	0	NA	NA	NA	0	
BW2	UBWC1	6/18/2024	2024	2024B	DT	P4	0	NA	NA	NA	0	
BW2	UBWC1	6/18/2024	2024	2024B	DT	P5	0	NA	NA	NA	0	
BW2	UBWC1	6/18/2024	2024	2024B	MT	P6	0	NA	NA	NA	0	

SiteN	WS	Date	Year	Ycat	Transect	Plot	pDen	pmnBL	pmnHCW	lgHCW	pOCR	
BW2	UBWC1	6/18/2024	2024	2024B	MT	P7	0	NA	NA	NA	0	
BW2	UBWC1	6/18/2024	2024	2024B	MT	P8	0	NA	NA	NA	0	
BW2	UBWC1	6/18/2024	2024	2024B	MT	P9	0	NA	NA	NA	0	
BW2	UBWC1	6/18/2024	2024	2024B	MT	P10	0	NA	NA	NA	0	
BW2	UBWC1	6/18/2024	2024	2024B	UT	P11	0	NA	NA	NA	0	
BW2	UBWC1	6/18/2024	2024	2024B	UT	P12	0	NA	NA	NA	0	
BW2	UBWC1	6/18/2024	2024	2024B	UT	P13	0	NA	NA	NA	0	
BW2	UBWC1	6/18/2024	2024	2024B	UT	P14	0	NA	NA	NA	0	
BW2	UBWC1	6/18/2024	2024	2024B	UT	P15	0	NA	NA	NA	0	
BW2	UBWC1	6/18/2024	2024	2024B	SS	P16	0	NA	NA	NA	0	
BW2	UBWC1	6/18/2024	2024	2024B	SS	P17	0	NA	NA	NA	0	
BW2	UBWC1	6/18/2024	2024	2024B	SS	P18	0	NA	NA	NA	0	
BW2	UBWC1	6/18/2024	2024	2024B	SS	P19	0	NA	NA	NA	0	
BW2	UBWC1	6/18/2024	2024	2024B	SS	P20	0	NA	NA	NA	0	
BW1	UBWC1	6/24/2024	2024	2024B	DT	P1	0	NA	NA	NA	0	
BW1	UBWC1	6/24/2024	2024	2024B	DT	P2	0	NA	NA	NA	0	
BW1	UBWC1	6/24/2024	2024	2024B	DT	P3	0	NA	NA	NA	0	
BW1	UBWC1	6/24/2024	2024	2024B	DT	P4	0	NA	NA	NA	0	
BW1	UBWC1	6/24/2024	2024	2024B	DT	P5	0	NA	NA	NA	0	
BW1	UBWC1	6/24/2024	2024	2024B	MT	P6	0	NA	NA	NA	0	
BW1	UBWC1	6/24/2024	2024	2024B	MT	P7	0	NA	NA	NA	0	
BW1	UBWC1	6/24/2024	2024	2024B	MT	P8	0	NA	NA	NA	0	
BW1	UBWC1	6/24/2024	2024	2024B	MT	P9	0	NA	NA	NA	0	
BW1	UBWC1	6/24/2024	2024	2024B	MT	P10	0	NA	NA	NA	0	
BW1	UBWC1	6/24/2024	2024	2024B	UT	P11	0	NA	NA	NA	0	
BW1	UBWC1	6/24/2024	2024	2024B	UT	P12	0	NA	NA	NA	0	
BW1	UBWC1	6/24/2024	2024	2024B	UT	P13	0	NA	NA	NA	0	
BW1	UBWC1	6/24/2024	2024	2024B	UT	P14	0	NA	NA	NA	0	
BW1	UBWC1	6/24/2024	2024	2024B	UT	P15	0	NA	NA	NA	0	
BW1	UBWC1	6/24/2024	2024	2024B	SS	P16	0	NA	NA	NA	0	
BW1	UBWC1	6/24/2024	2024	2024B	SS	P17	0	NA	NA	NA	0	

SiteN	WS	Date	Year	Ycat	Transect	Plot	pDen	pmnBL	pmnHCW	lgHCW	pOCR	
BW1	UBWC1	6/24/2024	2024	2024B	SS	P18	0	NA	NA	NA	0	
BW1	UBWC1	6/24/2024	2024	2024B	SS	P19	0	NA	NA	NA	0	
BW1	UBWC1	6/24/2024	2024	2024B	SS	P20	1	33	3.5	0.653	1	
CF1	MOHI1	7/2/2024	2024	2024B	DT	P1	0	NA	NA	NA	0	
CF1	MOHI1	7/2/2024	2024	2024B	DT	P2	0	NA	NA	NA	0	
CF1	MOHI1	7/2/2024	2024	2024B	DT	P3	2	43	3.5	0.653	1	
CF1	MOHI1	7/2/2024	2024	2024B	DT	P4	2	40.5	3.75	0.677	1	
CF1	MOHI1	7/2/2024	2024	2024B	DT	P5	0	NA	NA	NA	0	
CF1	MOHI1	7/2/2024	2024	2024B	MT	P6	2	47.5	4.5	0.74	1	
CF1	MOHI1	7/2/2024	2024	2024B	MT	P7	6	40.33	4.42	0.734	1	
CF1	MOHI1	7/2/2024	2024	2024B	MT	P8	8	37.31	3.88	0.688	1	
CF1	MOHI1	7/2/2024	2024	2024B	MT	P9	3	45	5.67	0.824	1	
CF1	MOHI1	7/2/2024	2024	2024B	MT	P10	0	NA	NA	NA	0	
CF1	MOHI1	7/2/2024	2024	2024B	UT	P11	0	NA	NA	NA	0	
CF1	MOHI1	7/2/2024	2024	2024B	UT	P12	0	NA	NA	NA	0	
CF1	MOHI1	7/2/2024	2024	2024B	UT	P13	0	NA	NA	NA	0	
CF1	MOHI1	7/2/2024	2024	2024B	UT	P14	0	NA	NA	NA	0	
CF1	MOHI1	7/2/2024	2024	2024B	UT	P15	0	NA	NA	NA	0	
CF1	MOHI1	7/2/2024	2024	2024B	SS	P16	1	44	4.5	0.74	1	
CF1	MOHI1	7/2/2024	2024	2024B	SS	P17	4	38	4.38	0.731	1	
CF1	MOHI1	7/2/2024	2024	2024B	SS	P18	3	38	3.83	0.684	1	
CF1	MOHI1	7/2/2024	2024	2024B	SS	P19	5	31.8	3.1	0.613	1	
CF1	MOHI1	7/2/2024	2024	2024B	SS	P20	10	61.9	7.05	0.906	1	
KR2	KOKO1	7/9/2024	2024	2024B	DT	P1	0	NA	NA	NA	0	
KR2	KOKO1	7/9/2024	2024	2024B	DT	P2	0	NA	NA	NA	0	
KR2	KOKO1	7/9/2024	2024	2024B	DT	P3	0	NA	NA	NA	0	
KR2	KOKO1	7/9/2024	2024	2024B	DT	P4	0	NA	NA	NA	0	
KR2	KOKO1	7/9/2024	2024	2024B	DT	P5	0	NA	NA	NA	0	
KR2	KOKO1	7/9/2024	2024	2024B	MT	P6	0	NA	NA	NA	0	
KR2	KOKO1	7/9/2024	2024	2024B	MT	P7	0	NA	NA	NA	0	
KR2	KOKO1	7/9/2024	2024	2024B	MT	P8	0	NA	NA	NA	0	

SiteN	WS	Date	Year	Ycat	Transect	Plot	pDen	pmnBL	pmnHCW	lgHCW	pOCR	
KR2	KOKO1	7/9/2024	2024	2024B	MT	P9	1	67	7.5	0.929	1	
KR2	KOKO1	7/9/2024	2024	2024B	MT	P10	0	NA	NA	NA	0	
KR2	KOKO1	7/9/2024	2024	2024B	UT	P11	0	NA	NA	NA	0	
KR2	KOKO1	7/9/2024	2024	2024B	UT	P12	0	NA	NA	NA	0	
KR2	KOKO1	7/9/2024	2024	2024B	UT	P13	0	NA	NA	NA	0	
KR2	KOKO1	7/9/2024	2024	2024B	UT	P14	0	NA	NA	NA	0	
KR2	KOKO1	7/9/2024	2024	2024B	UT	P15	0	NA	NA	NA	0	
KR2	KOKO1	7/9/2024	2024	2024B	SS	P16	0	NA	NA	NA	0	
KR2	KOKO1	7/9/2024	2024	2024B	SS	P17	0	NA	NA	NA	0	
KR2	KOKO1	7/9/2024	2024	2024B	SS	P18	0	NA	NA	NA	0	
KR2	KOKO1	7/9/2024	2024	2024B	SS	P19	0	NA	NA	NA	0	
KR2	KOKO1	7/9/2024	2024	2024B	SS	P20	0	NA	NA	NA	0	
**Part 2 - Plot level instream habitat variables**												
**SiteN**	**WS**	**Date**	**Year**	**Ycat**	**Transect**	**Plot**	**Wdep**	**Wvel**	**SubRich**	**perBOCO**	**GSScore**	**ocrLWD**
KR1	KOKO1	6/5/2023	2023	2023A	DT	P1	0.13	0.13	2	60	160	0
KR1	KOKO1	6/5/2023	2023	2023A	DT	P2	0.27	0.7	2	85	160	0
KR1	KOKO1	6/5/2023	2023	2023A	DT	P3	0.22	0.85	2	50	97	0
KR1	KOKO1	6/5/2023	2023	2023A	DT	P4	0.18	0.44	3	75	160	0
KR1	KOKO1	6/5/2023	2023	2023A	DT	P5	0.1	0.09	3	80	160	0
KR1	KOKO1	6/5/2023	2023	2023A	MT	P1	0.03	NA	2	35	33	0
KR1	KOKO1	6/5/2023	2023	2023A	MT	P2	0.1	0.5	2	40	33	0
KR1	KOKO1	6/5/2023	2023	2023A	MT	P3	0.15	0.83	2	90	160	0
KR1	KOKO1	6/5/2023	2023	2023A	MT	P4	0.2	0.73	2	50	97	0
KR1	KOKO1	6/5/2023	2023	2023A	MT	P5	0.2	0.42	3	90	160	0
KR1	KOKO1	6/5/2023	2023	2023A	UT	P1	0.13	0.37	2	10	33	0
KR1	KOKO1	6/5/2023	2023	2023A	UT	P2	0.2	0.64	2	20	33	0
KR1	KOKO1	6/5/2023	2023	2023A	UT	P3	0.1	0.89	2	50	33	0
KR1	KOKO1	6/5/2023	2023	2023A	UT	P4	0.15	0.39	2	20	33	0
KR1	KOKO1	6/5/2023	2023	2023A	UT	P5	0.25	0.2	2	40	33	0
KR1	KOKO1	6/5/2023	2023	2023A	SS	SS1	0.2	0.98	2	0	1	1

SiteN	WS	Date	Year	Ycat	Transect	Plot	Wdep	Wvel	SubRich	perBOCO	GSScore	ocrLWD
KR1	KOKO1	6/5/2023	2023	2023A	SS	SS2	0.28	0.73	3	90	160	0
KR1	KOKO1	6/5/2023	2023	2023A	SS	SS3	0.19	1.02	2	100	260	0
KR1	KOKO1	6/5/2023	2023	2023A	SS	SS4	0.11	0.68	3	75	160	0
KR1	KOKO1	6/5/2023	2023	2023A	SS	SS5	0.12	0.68	2	85	160	0
KR3	KOKO1	6/6/2023	2023	2023A	DT	P1	0.25	0.37	3	40	33	0
KR3	KOKO1	6/6/2023	2023	2023A	DT	P2	0.3	0.66	3	55	160	0
KR3	KOKO1	6/6/2023	2023	2023A	DT	P3	0.58	0.51	2	0	1	0
KR3	KOKO1	6/6/2023	2023	2023A	DT	P4	0.28	0.55	2	0	0	0
KR3	KOKO1	6/6/2023	2023	2023A	DT	P5	0.16	0.52	1	0	0	0
KR3	KOKO1	6/6/2023	2023	2023A	MT	P1	0.45	0.67	2	10	33	0
KR3	KOKO1	6/6/2023	2023	2023A	MT	P2	0.09	0.6	1	0	0	0
KR3	KOKO1	6/6/2023	2023	2023A	MT	P3	0.1	0.46	2	90	260	0
KR3	KOKO1	6/6/2023	2023	2023A	MT	P4	0.2	0.72	2	15	0	0
KR3	KOKO1	6/6/2023	2023	2023A	MT	P5	0.11	0.65	1	0	0	0
KR3	KOKO1	6/6/2023	2023	2023A	UT	P1	0.61	0.63	3	0	0	0
KR3	KOKO1	6/6/2023	2023	2023A	UT	P2	0.21	0.33	2	0	0	0
KR3	KOKO1	6/6/2023	2023	2023A	UT	P3	0.3	0.2	2	0	0	0
KR3	KOKO1	6/6/2023	2023	2023A	UT	P4	0.15	0.54	5	15	0	0
KR3	KOKO1	6/6/2023	2023	2023A	UT	P5	0.14	0.43	2	2	0	0
KR3	KOKO1	6/6/2023	2023	2023A	SS	SS1	0.22	0.55	4	30	260	0
KR3	KOKO1	6/6/2023	2023	2023A	SS	SS2	0.1	0.57	3	95	260	0
KR3	KOKO1	6/6/2023	2023	2023A	SS	SS3	0.19	0.49	4	25	0	0
KR3	KOKO1	6/6/2023	2023	2023A	SS	SS4	0.2	0.39	5	60	260	0
KR3	KOKO1	6/6/2023	2023	2023A	SS	SS5	0.16	0.71	2	85	260	0
AC1	ALUM1	6/29/2023	2023	2023A	DT	P1	0.03	NA	3	5	33	0
AC1	ALUM1	6/29/2023	2023	2023A	DT	P2	0.1	0.05	3	20	17	0
AC1	ALUM1	6/29/2023	2023	2023A	DT	P3	0.19	0.2	2	45	33	0
AC1	ALUM1	6/29/2023	2023	2023A	DT	P4	0.39	0.45	2	5	0	0
AC1	ALUM1	6/29/2023	2023	2023A	DT	P5	0.3	0.06	2	5	0	0
AC1	ALUM1	6/29/2023	2023	2023A	MT	P1	0.05	0.2	3	75	160	0
AC1	ALUM1	6/29/2023	2023	2023A	MT	P2	0.1	0.48	3	80	160	0

SiteN	WS	Date	Year	Ycat	Transect	Plot	Wdep	Wvel	SubRich	perBOCO	GSScore	ocrLWD
AC1	ALUM1	6/29/2023	2023	2023A	MT	P3	0.1	0.3	3	60	147	0
AC1	ALUM1	6/29/2023	2023	2023A	MT	P4	0.2	0.42	3	85	260	0
AC1	ALUM1	6/29/2023	2023	2023A	MT	P5	0.26	0.82	3	80	260	0
AC1	ALUM1	6/29/2023	2023	2023A	UT	P1	0.18	0.05	1	0	0	0
AC1	ALUM1	6/29/2023	2023	2023A	UT	P2	0.28	0.05	2	0	33	0
AC1	ALUM1	6/29/2023	2023	2023A	UT	P3	0.3	0.12	2	0	0	0
AC1	ALUM1	6/29/2023	2023	2023A	UT	P4	0.33	0.23	1	0	0	0
AC1	ALUM1	6/29/2023	2023	2023A	UT	P5	0.33	0.06	3	60	160	0
AC1	ALUM1	6/29/2023	2023	2023A	SS	SS1	0.27	0.27	2	40	0	0
AC1	ALUM1	6/29/2023	2023	2023A	SS	SS2	0.16	0.33	3	65	260	0
AC1	ALUM1	6/29/2023	2023	2023A	SS	SS3	0.22	1.02	1	100	260	0
AC1	ALUM1	6/29/2023	2023	2023A	SS	SS4	0.2	0.58	2	90	260	0
AC1	ALUM1	6/29/2023	2023	2023A	SS	SS5	0.15	0.66	2	80	260	0
AC2	ALUM1	6/29/2023	2023	2023A	DT	P1	0.2	0.3	1	0	0	0
AC2	ALUM1	6/29/2023	2023	2023A	DT	P2	0.26	0.25	2	0	0	0
AC2	ALUM1	6/29/2023	2023	2023A	DT	P3	0.2	0.24	4	80	260	0
AC2	ALUM1	6/29/2023	2023	2023A	DT	P4	0.1	0.18	2	5	33	0
AC2	ALUM1	6/29/2023	2023	2023A	DT	P5	0.2	0.24	3	50	160	0
AC2	ALUM1	6/29/2023	2023	2023A	MT	P1	0.12	0.56	2	85	160	0
AC2	ALUM1	6/29/2023	2023	2023A	MT	P2	0.18	0.36	2	85	160	0
AC2	ALUM1	6/29/2023	2023	2023A	MT	P3	0.2	0.4	3	90	160	0
AC2	ALUM1	6/29/2023	2023	2023A	MT	P4	0.16	0.36	4	75	160	0
AC2	ALUM1	6/29/2023	2023	2023A	MT	P5	0.06	0.15	2	90	160	0
AC2	ALUM1	6/29/2023	2023	2023A	UT	P1	0.1	0.6	3	5	33	0
AC2	ALUM1	6/29/2023	2023	2023A	UT	P2	0.1	0.34	3	95	260	0
AC2	ALUM1	6/29/2023	2023	2023A	UT	P3	0.1	0.2	3	95	160	0
AC2	ALUM1	6/29/2023	2023	2023A	UT	P4	0.07	0.06	4	45	1	0
AC2	ALUM1	6/29/2023	2023	2023A	UT	P5	0.2	0	4	90	260	0
AC2	ALUM1	6/29/2023	2023	2023A	SS	SS1	0.15	0.78	2	55	160	0
AC2	ALUM1	6/29/2023	2023	2023A	SS	SS2	0.12	0.92	2	60	160	0
AC2	ALUM1	6/29/2023	2023	2023A	SS	SS3	0.15	0.7	2	40	0	0

SiteN	WS	Date	Year	Ycat	Transect	Plot	Wdep	Wvel	SubRich	perBOCO	GSScore	ocrLWD
AC2	ALUM1	6/29/2023	2023	2023A	SS	SS4	0.15	1.09	1	100	160	0
AC2	ALUM1	6/29/2023	2023	2023A	SS	SS5	0.12	0.95	2	80	160	0
CF3	MOHI1	7/7/2023	2023	2023A	DT	P1	0.34	0.62	3	80	210	0
CF3	MOHI1	7/7/2023	2023	2023A	DT	P2	0.46	1.02	2	90	160	0
CF3	MOHI1	7/7/2023	2023	2023A	DT	P3	0.43	0.75	2	90	160	0
CF3	MOHI1	7/7/2023	2023	2023A	DT	P4	0.09	0.38	3	95	210	0
CF3	MOHI1	7/7/2023	2023	2023A	DT	P5	0.12	0.32	2	85	160	0
CF3	MOHI1	7/7/2023	2023	2023A	MT	P1	0.37	0.66	2	75	160	0
CF3	MOHI1	7/7/2023	2023	2023A	MT	P2	0.41	0.81	3	80	160	0
CF3	MOHI1	7/7/2023	2023	2023A	MT	P3	0.32	1.13	3	80	160	0
CF3	MOHI1	7/7/2023	2023	2023A	MT	P4	0.15	0.55	2	90	160	0
CF3	MOHI1	7/7/2023	2023	2023A	MT	P5	0.06	0.26	2	85	160	0
CF3	MOHI1	7/7/2023	2023	2023A	UT	P1	0.46	0.62	2	75	160	0
CF3	MOHI1	7/7/2023	2023	2023A	UT	P2	0.34	0.12	4	75	160	0
CF3	MOHI1	7/7/2023	2023	2023A	UT	P3	0.7	0.87	1	100	160	0
CF3	MOHI1	7/7/2023	2023	2023A	UT	P4	0.14	0.49	3	85	160	0
CF3	MOHI1	7/7/2023	2023	2023A	UT	P5	0.03	0.28	2	15	33	0
CF3	MOHI1	7/7/2023	2023	2023A	SS	SS1	0.34	0.85	2	100	260	0
CF3	MOHI1	7/7/2023	2023	2023A	SS	SS2	0.08	0.45	2	90	160	0
CF3	MOHI1	7/7/2023	2023	2023A	SS	SS3	0.09	1.07	2	100	260	0
CF3	MOHI1	7/7/2023	2023	2023A	SS	SS4	0.27	0.5	2	100	260	0
CF3	MOHI1	7/7/2023	2023	2023A	SS	SS5	0.3	0.71	2	100	260	0
CF2	MOHI1	7/8/2023	2023	2023A	DT	P1	0.27	0.3	3	90	160	0
CF2	MOHI1	7/8/2023	2023	2023A	DT	P2	0.43	0.58	2	80	160	0
CF2	MOHI1	7/8/2023	2023	2023A	DT	P3	0.43	0.7	2	80	160	0
CF2	MOHI1	7/8/2023	2023	2023A	DT	P4	0.46	0.58	3	40	160	0
CF2	MOHI1	7/8/2023	2023	2023A	DT	P5	0.46	0.07	2	0	1	0
CF2	MOHI1	7/8/2023	2023	2023A	MT	P1	0.24	0.53	2	60	160	0
CF2	MOHI1	7/8/2023	2023	2023A	MT	P2	0.43	0.53	2	80	160	0
CF2	MOHI1	7/8/2023	2023	2023A	MT	P3	0.38	0.63	2	80	160	0
CF2	MOHI1	7/8/2023	2023	2023A	MT	P4	0.29	0.65	3	90	160	0

SiteN	WS	Date	Year	Ycat	Transect	Plot	Wdep	Wvel	SubRich	perBOCO	GSScore	ocrLWD
CF2	MOHI1	7/8/2023	2023	2023A	MT	P5	0.26	0.54	2	80	160	0
CF2	MOHI1	7/8/2023	2023	2023A	UT	P1	0.09	0.19	3	60	97	0
CF2	MOHI1	7/8/2023	2023	2023A	UT	P2	0.23	0.69	3	40	33	0
CF2	MOHI1	7/8/2023	2023	2023A	UT	P3	0.24	0.41	3	60	33	0
CF2	MOHI1	7/8/2023	2023	2023A	UT	P4	0.21	0.49	2	80	160	0
CF2	MOHI1	7/8/2023	2023	2023A	UT	P5	0.24	0.39	2	80	160	0
CF2	MOHI1	7/8/2023	2023	2023A	SS	SS1	0.38	0.78	3	80	260	0
CF2	MOHI1	7/8/2023	2023	2023A	SS	SS2	0.35	0.55	4	80	260	0
CF2	MOHI1	7/8/2023	2023	2023A	SS	SS3	0.27	0.88	2	100	260	0
CF2	MOHI1	7/8/2023	2023	2023A	SS	SS4	0.29	0.72	4	80	210	0
CF2	MOHI1	7/8/2023	2023	2023A	SS	SS5	0.3	0.77	3	90	260	0
BW2	UBWC1	7/11/2023	2023	2023A	DT	P1	0.23	0.24	3	85	160	0
BW2	UBWC1	7/11/2023	2023	2023A	DT	P2	0.18	0.5	3	85	160	0
BW2	UBWC1	7/11/2023	2023	2023A	DT	P3	0.15	0.41	2	85	160	0
BW2	UBWC1	7/11/2023	2023	2023A	DT	P4	0.18	0.43	2	85	160	0
BW2	UBWC1	7/11/2023	2023	2023A	DT	P5	0.15	0.08	3	85	260	0
BW2	UBWC1	7/11/2023	2023	2023A	MT	P1	0.14	0.35	3	90	160	0
BW2	UBWC1	7/11/2023	2023	2023A	MT	P2	0.18	0.42	4	80	210	0
BW2	UBWC1	7/11/2023	2023	2023A	MT	P3	0.21	0.4	2	90	160	0
BW2	UBWC1	7/11/2023	2023	2023A	MT	P4	0.14	0.34	2	90	160	0
BW2	UBWC1	7/11/2023	2023	2023A	MT	P5	0.11	0.15	2	75	160	0
BW2	UBWC1	7/11/2023	2023	2023A	UT	P1	0.06	0.37	3	80	210	0
BW2	UBWC1	7/11/2023	2023	2023A	UT	P2	0.2	0.64	3	80	210	0
BW2	UBWC1	7/11/2023	2023	2023A	UT	P3	0.17	0.32	2	100	160	0
BW2	UBWC1	7/11/2023	2023	2023A	UT	P4	0.21	0.32	2	100	160	0
BW2	UBWC1	7/11/2023	2023	2023A	UT	P5	0.14	0.22	4	70	160	0
BW2	UBWC1	7/11/2023	2023	2023A	SS	SS1	0.12	0.63	4	70	260	0
BW2	UBWC1	7/11/2023	2023	2023A	SS	SS2	0.11	0.22	4	80	160	0
BW2	UBWC1	7/11/2023	2023	2023A	SS	SS3	0.15	0.24	3	85	160	0
BW2	UBWC1	7/11/2023	2023	2023A	SS	SS4	0.12	0.27	3	90	260	0
BW2	UBWC1	7/11/2023	2023	2023A	SS	SS5	0.08	0.36	3	75	260	0

SiteN	WS	Date	Year	Ycat	Transect	Plot	Wdep	Wvel	SubRich	perBOCO	GSScore	ocrLWD
BW1	UBWC1	7/11/2023	2023	2023A	DT	P1	0.2	0.23	2	100	260	0
BW1	UBWC1	7/11/2023	2023	2023A	DT	P2	0.18	0.27	3	70	260	0
BW1	UBWC1	7/11/2023	2023	2023A	DT	P3	0.09	0.01	2	80	160	0
BW1	UBWC1	7/11/2023	2023	2023A	DT	P4	0.15	0.3	3	60	160	0
BW1	UBWC1	7/11/2023	2023	2023A	DT	P5	0.2	0.14	3	35	81	0
BW1	UBWC1	7/11/2023	2023	2023A	MT	P1	0.3	0.19	2	100	160	0
BW1	UBWC1	7/11/2023	2023	2023A	MT	P2	0.37	0.35	2	90	160	0
BW1	UBWC1	7/11/2023	2023	2023A	MT	P3	0.37	0.18	2	90	160	0
BW1	UBWC1	7/11/2023	2023	2023A	MT	P4	0.12	0.21	2	75	160	0
BW1	UBWC1	7/11/2023	2023	2023A	MT	P5	0.11	0.14	3	85	160	0
BW1	UBWC1	7/11/2023	2023	2023A	UT	P1	0.03	0.1	3	90	160	0
BW1	UBWC1	7/11/2023	2023	2023A	UT	P2	0.21	0.43	3	90	160	0
BW1	UBWC1	7/11/2023	2023	2023A	UT	P3	0.24	0.53	3	80	210	0
BW1	UBWC1	7/11/2023	2023	2023A	UT	P4	0.15	0.57	3	90	160	0
BW1	UBWC1	7/11/2023	2023	2023A	UT	P5	0.06	0.06	3	85	260	0
BW1	UBWC1	7/11/2023	2023	2023A	SS	SS1	0.09	0.55	3	90	160	0
BW1	UBWC1	7/11/2023	2023	2023A	SS	SS2	0.09	0.46	2	95	160	0
BW1	UBWC1	7/11/2023	2023	2023A	SS	SS3	0.17	0.39	3	90	260	0
BW1	UBWC1	7/11/2023	2023	2023A	SS	SS4	0.14	0.13	1	100	160	0
BW1	UBWC1	7/11/2023	2023	2023A	SS	SS5	0.21	0.73	3	80	160	0
CF1	MOHI1	7/19/2023	2023	2023A	DT	P1	0.09	0.3	2	10	33	0
CF1	MOHI1	7/19/2023	2023	2023A	DT	P2	0.29	0.87	1	100	260	0
CF1	MOHI1	7/19/2023	2023	2023A	DT	P3	0.46	0.64	2	0	0	0
CF1	MOHI1	7/19/2023	2023	2023A	DT	P4	0.4	0.23	4	40	0	0
CF1	MOHI1	7/19/2023	2023	2023A	DT	P5	0.15	0.37	2	90	160	0
CF1	MOHI1	7/19/2023	2023	2023A	MT	P1	0.15	0.21	4	70	260	0
CF1	MOHI1	7/19/2023	2023	2023A	MT	P2	0.21	0.26	3	80	160	0
CF1	MOHI1	7/19/2023	2023	2023A	MT	P3	0.32	0.82	2	40	33	0
CF1	MOHI1	7/19/2023	2023	2023A	MT	P4	0.2	0.53	2	0	33	0
CF1	MOHI1	7/19/2023	2023	2023A	MT	P5	0.05	-0.02	2	80	160	0
CF1	MOHI1	7/19/2023	2023	2023A	UT	P1	0.12	0.12	2	100	260	0

SiteN	WS	Date	Year	Ycat	Transect	Plot	Wdep	Wvel	SubRich	perBOCO	GSScore	ocrLWD
CF1	MOHI1	7/19/2023	2023	2023A	UT	P2	0.2	0.48	2	100	260	0
CF1	MOHI1	7/19/2023	2023	2023A	UT	P3	0.58	1.27	3	70	260	0
CF1	MOHI1	7/19/2023	2023	2023A	UT	P4	0.15	0.53	1	100	160	0
CF1	MOHI1	7/19/2023	2023	2023A	UT	P5	0.11	0.34	1	100	160	0
CF1	MOHI1	7/19/2023	2023	2023A	SS	SS1	0.2	0.76	3	90	260	0
CF1	MOHI1	7/19/2023	2023	2023A	SS	SS2	0.3	0.59	2	100	260	0
CF1	MOHI1	7/19/2023	2023	2023A	SS	SS3	0.27	1.04	2	100	260	0
CF1	MOHI1	7/19/2023	2023	2023A	SS	SS4	0.29	1.05	3	70	260	0
CF1	MOHI1	7/19/2023	2023	2023A	SS	SS5	0.43	0.82	2	100	160	0
KR1	KOKO1	6/4/2024	2024	2024B	DT	P1	0.06	0.09	3	90	160	0
KR1	KOKO1	6/4/2024	2024	2024B	DT	P2	0.15	0.32	2	90	160	0
KR1	KOKO1	6/4/2024	2024	2024B	DT	P3	0.24	0.88	2	90	160	0
KR1	KOKO1	6/4/2024	2024	2024B	DT	P4	0.21	0.31	2	80	160	0
KR1	KOKO1	6/4/2024	2024	2024B	DT	P5	0.12	0.21	4	80	160	0
KR1	KOKO1	6/4/2024	2024	2024B	MT	P6	0.05	0.04	3	85	160	0
KR1	KOKO1	6/4/2024	2024	2024B	MT	P7	0.09	0.31	2	80	160	0
KR1	KOKO1	6/4/2024	2024	2024B	MT	P8	0.17	0.45	2	80	160	0
KR1	KOKO1	6/4/2024	2024	2024B	MT	P9	0.19	0.69	2	60	160	0
KR1	KOKO1	6/4/2024	2024	2024B	MT	P10	0.14	0.41	2	60	160	0
KR1	KOKO1	6/4/2024	2024	2024B	UT	P11	0.2	0.34	2	50	97	0
KR1	KOKO1	6/4/2024	2024	2024B	UT	P12	0.15	0.47	3	20	33	0
KR1	KOKO1	6/4/2024	2024	2024B	UT	P13	0.1	0.6	3	30	33	0
KR1	KOKO1	6/4/2024	2024	2024B	UT	P14	0.08	0.05	3	45	81	0
KR1	KOKO1	6/4/2024	2024	2024B	UT	P15	0.17	0.2	3	40	81	0
KR1	KOKO1	6/4/2024	2024	2024B	SS	P16	0.26	0.49	3	80	160	0
KR1	KOKO1	6/4/2024	2024	2024B	SS	P17	0.27	0.81	3	90	210	0
KR1	KOKO1	6/4/2024	2024	2024B	SS	P18	0.18	0.45	3	90	210	0
KR1	KOKO1	6/4/2024	2024	2024B	SS	P19	0.16	0.63	3	75	260	0
KR1	KOKO1	6/4/2024	2024	2024B	SS	P20	0.12	0.47	3	90	160	0
KR3	KOKO1	6/25/2024	2024	2024B	DT	P1	0.07	0.11	3	20	1	0
KR3	KOKO1	6/25/2024	2024	2024B	DT	P2	0.05	0.32	3	50	160	0

SiteN	WS	Date	Year	Ycat	Transect	Plot	Wdep	Wvel	SubRich	perBOCO	GSScore	ocrLWD
KR3	KOKO1	6/25/2024	2024	2024B	DT	P3	0.34	0.32	3	20	17	0
KR3	KOKO1	6/25/2024	2024	2024B	DT	P4	0.54	0.38	1	0	0	0
KR3	KOKO1	6/25/2024	2024	2024B	DT	P5	0.26	0.04	3	85	160	0
KR3	KOKO1	6/25/2024	2024	2024B	MT	P6	0.23	0.78	3	60	160	0
KR3	KOKO1	6/25/2024	2024	2024B	MT	P7	0.22	0.42	4	30	0	0
KR3	KOKO1	6/25/2024	2024	2024B	MT	P8	0.24	0.15	4	10	0	0
KR3	KOKO1	6/25/2024	2024	2024B	MT	P9	0.24	0.62	3	0	0	0
KR3	KOKO1	6/25/2024	2024	2024B	MT	P10	0.14	0.56	3	0	0	0
KR3	KOKO1	6/25/2024	2024	2024B	UT	P11	0.52	0.7	4	5	0	0
KR3	KOKO1	6/25/2024	2024	2024B	UT	P12	0.31	0.11	4	10	0	0
KR3	KOKO1	6/25/2024	2024	2024B	UT	P13	0.19	0.37	3	0	0	0
KR3	KOKO1	6/25/2024	2024	2024B	UT	P14	0.09	0.44	2	0	0	0
KR3	KOKO1	6/25/2024	2024	2024B	UT	P15	0.14	0.91	4	5	0	0
KR3	KOKO1	6/25/2024	2024	2024B	SS	P16	0.28	0.52	4	80	210	0
KR3	KOKO1	6/25/2024	2024	2024B	SS	P17	0.12	0.92	5	45	0	0
KR3	KOKO1	6/25/2024	2024	2024B	SS	P18	0.16	0.58	4	75	210	0
KR3	KOKO1	6/25/2024	2024	2024B	SS	P19	0.16	0.5	5	55	210	0
KR3	KOKO1	6/25/2024	2024	2024B	SS	P20	0.21	0.48	5	55	130	0
AC1	ALUM1	6/10/2024	2024	2024B	DT	P1	0.03	0.01	2	50	97	0
AC1	ALUM1	6/10/2024	2024	2024B	DT	P2	0.03	0.01	2	20	33	0
AC1	ALUM1	6/10/2024	2024	2024B	DT	P3	0.23	0.1	3	90	97	0
AC1	ALUM1	6/10/2024	2024	2024B	DT	P4	0.35	0.47	4	15	0	0
AC1	ALUM1	6/10/2024	2024	2024B	DT	P5	0.26	0.11	2	5	0	0
AC1	ALUM1	6/10/2024	2024	2024B	MT	P6	0.05	0.12	3	25	33	0
AC1	ALUM1	6/10/2024	2024	2024B	MT	P7	0.03	0.12	3	70	160	0
AC1	ALUM1	6/10/2024	2024	2024B	MT	P8	0.11	0.44	4	90	260	0
AC1	ALUM1	6/10/2024	2024	2024B	MT	P9	0.15	0.58	4	90	260	0
AC1	ALUM1	6/10/2024	2024	2024B	MT	P10	0.21	0.03	2	100	260	0
AC1	ALUM1	6/10/2024	2024	2024B	UT	P11	0.14	0	3	0	1	0
AC1	ALUM1	6/10/2024	2024	2024B	UT	P12	0.26	0.02	3	0	0.5	0
AC1	ALUM1	6/10/2024	2024	2024B	UT	P13	0.29	0.18	2	0	0	0

SiteN	WS	Date	Year	Ycat	Transect	Plot	Wdep	Wvel	SubRich	perBOCO	GSScore	ocrLWD
AC1	ALUM1	6/10/2024	2024	2024B	UT	P14	0.38	0.18	4	50	0	0
AC1	ALUM1	6/10/2024	2024	2024B	UT	P15	0.23	0.05	4	30	0	0
AC1	ALUM1	6/10/2024	2024	2024B	SS	P16	0.14	0.66	4	90	260	0
AC1	ALUM1	6/10/2024	2024	2024B	SS	P17	0.11	0.39	2	100	260	0
AC1	ALUM1	6/10/2024	2024	2024B	SS	P18	0.15	0.25	2	100	260	0
AC1	ALUM1	6/10/2024	2024	2024B	SS	P19	0.23	0.34	2	100	260	0
AC1	ALUM1	6/10/2024	2024	2024B	SS	P20	0.16	0.58	3	95	260	0
AC2	ALUM1	6/11/2024	2024	2024B	DT	P1	0.12	0.08	4	30	0	0
AC2	ALUM1	6/11/2024	2024	2024B	DT	P2	0.17	0.18	3	0	0	0
AC2	ALUM1	6/11/2024	2024	2024B	DT	P3	0.23	0.28	4	65	260	0
AC2	ALUM1	6/11/2024	2024	2024B	DT	P4	0.12	0.01	2	90	160	0
AC2	ALUM1	6/11/2024	2024	2024B	DT	P5	0.1	0.08	3	90	160	0
AC2	ALUM1	6/11/2024	2024	2024B	MT	P6	0.09	0.06	3	20	33	0
AC2	ALUM1	6/11/2024	2024	2024B	MT	P7	0.07	0.04	4	50	260	0
AC2	ALUM1	6/11/2024	2024	2024B	MT	P8	0.13	0.06	3	5	33	0
AC2	ALUM1	6/11/2024	2024	2024B	MT	P9	0.23	0.1	3	90	260	0
AC2	ALUM1	6/11/2024	2024	2024B	MT	P10	0.18	0.03	4	60	160	0
AC2	ALUM1	6/11/2024	2024	2024B	UT	P11	0.14	0.25	4	35	33	0
AC2	ALUM1	6/11/2024	2024	2024B	UT	P12	0.08	0.08	4	50	160	0
AC2	ALUM1	6/11/2024	2024	2024B	UT	P13	0.07	0.06	3	50	160	0
AC2	ALUM1	6/11/2024	2024	2024B	UT	P14	0.16	0.03	3	90	160	0
AC2	ALUM1	6/11/2024	2024	2024B	UT	P15	0.36	0.03	2	20	0	0
AC2	ALUM1	6/11/2024	2024	2024B	SS	P16	0.26	0.55	5	50	260	0
AC2	ALUM1	6/11/2024	2024	2024B	SS	P17	0.08	0.27	3	90	160	0
AC2	ALUM1	6/11/2024	2024	2024B	SS	P18	0.18	0.26	3	95	160	0
AC2	ALUM1	6/11/2024	2024	2024B	SS	P19	0.12	0.74	3	95	160	0
AC2	ALUM1	6/11/2024	2024	2024B	SS	P20	0.15	1.14	3	60	33	0
CF3	MOHI1	6/17/2024	2024	2024B	DT	P1	0.27	0.48	2	100	160	0
CF3	MOHI1	6/17/2024	2024	2024B	DT	P2	0.32	0.77	3	90	160	0
CF3	MOHI1	6/17/2024	2024	2024B	DT	P3	0.35	0.83	4	75	160	0
CF3	MOHI1	6/17/2024	2024	2024B	DT	P4	0.17	0.01	3	50	160	0

SiteN	WS	Date	Year	Ycat	Transect	Plot	Wdep	Wvel	SubRich	perBOCO	GSScore	ocrLWD
CF3	MOHI1	6/17/2024	2024	2024B	DT	P5	0.12	0.01	3	65	160	0
CF3	MOHI1	6/17/2024	2024	2024B	MT	P6	0.27	0.52	3	35	33	0
CF3	MOHI1	6/17/2024	2024	2024B	MT	P7	0.35	0.62	3	50	160	0
CF3	MOHI1	6/17/2024	2024	2024B	MT	P8	0.27	0.81	3	80	160	0
CF3	MOHI1	6/17/2024	2024	2024B	MT	P9	0.14	0.32	3	70	160	0
CF3	MOHI1	6/17/2024	2024	2024B	MT	P10	0.06	0.05	2	75	160	0
CF3	MOHI1	6/17/2024	2024	2024B	UT	P11	0.29	0.04	4	90	260	0
CF3	MOHI1	6/17/2024	2024	2024B	UT	P12	0.25	0.12	4	90	260	0
CF3	MOHI1	6/17/2024	2024	2024B	UT	P13	0.4	0.54	4	80	260	0
CF3	MOHI1	6/17/2024	2024	2024B	UT	P14	0.39	0.54	4	65	260	0
CF3	MOHI1	6/17/2024	2024	2024B	UT	P15	0.27	0.23	3	60	160	0
CF3	MOHI1	6/17/2024	2024	2024B	SS	P16	0.16	0.8	3	90	260	0
CF3	MOHI1	6/17/2024	2024	2024B	SS	P17	0.32	0.85	3	90	260	0
CF3	MOHI1	6/17/2024	2024	2024B	SS	P18	0.42	0.35	4	85	260	0
CF3	MOHI1	6/17/2024	2024	2024B	SS	P19	0.36	0.21	3	90	160	0
CF3	MOHI1	6/17/2024	2024	2024B	SS	P20	0.2	0.08	2	95	160	0
CF2	MOHI1	7/1/2024	2024	2024B	DT	P1	0.23	0.43	2	85	160	0
CF2	MOHI1	7/1/2024	2024	2024B	DT	P2	0.37	0.66	3	95	160	0
CF2	MOHI1	7/1/2024	2024	2024B	DT	P3	0.28	0.79	2	60	160	0
CF2	MOHI1	7/1/2024	2024	2024B	DT	P4	0.21	0.76	2	75	160	0
CF2	MOHI1	7/1/2024	2024	2024B	DT	P5	0.16	0.43	2	40	33	0
CF2	MOHI1	7/1/2024	2024	2024B	MT	P6	0.15	0.51	3	90	160	0
CF2	MOHI1	7/1/2024	2024	2024B	MT	P7	0.31	0.57	2	85	160	0
CF2	MOHI1	7/1/2024	2024	2024B	MT	P8	0.27	0.89	3	95	160	0
CF2	MOHI1	7/1/2024	2024	2024B	MT	P9	0.21	0.98	3	80	160	0
CF2	MOHI1	7/1/2024	2024	2024B	MT	P10	0.14	0.41	3	75	160	0
CF2	MOHI1	7/1/2024	2024	2024B	UT	P11	0.09	0.21	3	85	160	0
CF2	MOHI1	7/1/2024	2024	2024B	UT	P12	0.18	0.42	3	60	147	0
CF2	MOHI1	7/1/2024	2024	2024B	UT	P13	0.21	0.35	4	50	147	0
CF2	MOHI1	7/1/2024	2024	2024B	UT	P14	0.23	0.36	3	30	17	0
CF2	MOHI1	7/1/2024	2024	2024B	UT	P15	0.24	0.42	3	30	33	0

SiteN	WS	Date	Year	Ycat	Transect	Plot	Wdep	Wvel	SubRich	perBOCO	GSScore	ocrLWD
CF2	MOHI1	7/1/2024	2024	2024B	SS	P16	0.26	0.68	2	100	210	0
CF2	MOHI1	7/1/2024	2024	2024B	SS	P17	0.3	0.69	3	90	260	0
CF2	MOHI1	7/1/2024	2024	2024B	SS	P18	0.3	0.63	3	90	260	0
CF2	MOHI1	7/1/2024	2024	2024B	SS	P19	0.24	0.94	3	90	260	0
CF2	MOHI1	7/1/2024	2024	2024B	SS	P20	0.2	0.73	3	90	260	0
BW2	UBWC1	6/18/2024	2024	2024B	DT	P1	0.11	0.03	4	70	260	0
BW2	UBWC1	6/18/2024	2024	2024B	DT	P2	0.12	0	4	55	160	0
BW2	UBWC1	6/18/2024	2024	2024B	DT	P3	0.12	0.02	2	100	260	0
BW2	UBWC1	6/18/2024	2024	2024B	DT	P4	0.24	0.2	2	70	160	0
BW2	UBWC1	6/18/2024	2024	2024B	DT	P5	0.17	0.13	4	70	160	0
BW2	UBWC1	6/18/2024	2024	2024B	MT	P6	0.15	0.14	3	80	160	0
BW2	UBWC1	6/18/2024	2024	2024B	MT	P7	0.1	0.16	3	90	260	0
BW2	UBWC1	6/18/2024	2024	2024B	MT	P8	0.16	0.19	3	95	160	0
BW2	UBWC1	6/18/2024	2024	2024B	MT	P9	0.16	0.13	3	80	160	0
BW2	UBWC1	6/18/2024	2024	2024B	MT	P10	0.11	0.32	4	70	160	0
BW2	UBWC1	6/18/2024	2024	2024B	UT	P11	0.17	0.14	4	85	160	0
BW2	UBWC1	6/18/2024	2024	2024B	UT	P12	0.27	0.08	3	85	260	0
BW2	UBWC1	6/18/2024	2024	2024B	UT	P13	0.24	0.21	3	80	160	0
BW2	UBWC1	6/18/2024	2024	2024B	UT	P14	0.11	0.17	3	90	160	0
BW2	UBWC1	6/18/2024	2024	2024B	UT	P15	0.05	0.17	4	80	210	0
BW2	UBWC1	6/18/2024	2024	2024B	SS	P16	0.13	0.1	4	85	260	0
BW2	UBWC1	6/18/2024	2024	2024B	SS	P17	0.1	0.23	3	90	260	0
BW2	UBWC1	6/18/2024	2024	2024B	SS	P18	0.12	0.46	3	90	260	0
BW2	UBWC1	6/18/2024	2024	2024B	SS	P19	0.09	0.76	3	90	260	0
BW2	UBWC1	6/18/2024	2024	2024B	SS	P20	0.12	0.38	3	85	160	0
BW1	UBWC1	6/24/2024	2024	2024B	DT	P1	0.22	0.16	4	70	260	0
BW1	UBWC1	6/24/2024	2024	2024B	DT	P2	0.16	0.07	4	90	160	0
BW1	UBWC1	6/24/2024	2024	2024B	DT	P3	0.17	0.16	4	90	260	0
BW1	UBWC1	6/24/2024	2024	2024B	DT	P4	0.27	0.05	3	30	33	0
BW1	UBWC1	6/24/2024	2024	2024B	DT	P5	0.38	0	3	40	81	1
BW1	UBWC1	6/24/2024	2024	2024B	MT	P6	0.21	0.04	3	78	160	0

SiteN	WS	Date	Year	Ycat	Transect	Plot	Wdep	Wvel	SubRich	perBOCO	GSScore	ocrLWD
BW1	UBWC1	6/24/2024	2024	2024B	MT	P7	0.19	0.09	4	60	160	0
BW1	UBWC1	6/24/2024	2024	2024B	MT	P8	0.2	0.16	4	60	160	0
BW1	UBWC1	6/24/2024	2024	2024B	MT	P9	0.14	0.06	3	95	260	0
BW1	UBWC1	6/24/2024	2024	2024B	MT	P10	0.06	0	4	89	260	0
BW1	UBWC1	6/24/2024	2024	2024B	UT	P11	0.04	0.06	4	80	160	0
BW1	UBWC1	6/24/2024	2024	2024B	UT	P12	0.15	0.08	4	80	260	0
BW1	UBWC1	6/24/2024	2024	2024B	UT	P13	0.14	0.15	4	70	160	0
BW1	UBWC1	6/24/2024	2024	2024B	UT	P14	0.09	0.35	3	50	160	0
BW1	UBWC1	6/24/2024	2024	2024B	UT	P15	0.09	0.12	4	60	160	0
BW1	UBWC1	6/24/2024	2024	2024B	SS	P16	0.07	0.44	3	80	160	0
BW1	UBWC1	6/24/2024	2024	2024B	SS	P17	0.12	0.32	3	85	260	0
BW1	UBWC1	6/24/2024	2024	2024B	SS	P18	0.15	0.36	4	80	260	0
BW1	UBWC1	6/24/2024	2024	2024B	SS	P19	0.12	0.24	3	90	160	0
BW1	UBWC1	6/24/2024	2024	2024B	SS	P20	0.16	0.94	4	65	160	0
CF1	MOHI1	7/2/2024	2024	2024B	DT	P1	0.23	0.2	3	95	160	0
CF1	MOHI1	7/2/2024	2024	2024B	DT	P2	0.27	0.24	4	90	160	0
CF1	MOHI1	7/2/2024	2024	2024B	DT	P3	0.32	0.29	3	20	0	0
CF1	MOHI1	7/2/2024	2024	2024B	DT	P4	0.36	0.36	3	90	160	0
CF1	MOHI1	7/2/2024	2024	2024B	DT	P5	0.12	0.02	3	45	160	0
CF1	MOHI1	7/2/2024	2024	2024B	MT	P6	0.12	0.25	3	90	160	0
CF1	MOHI1	7/2/2024	2024	2024B	MT	P7	0.21	0.43	4	85	160	0
CF1	MOHI1	7/2/2024	2024	2024B	MT	P8	0.3	0.97	2	80	160	0
CF1	MOHI1	7/2/2024	2024	2024B	MT	P9	0.2	0.95	3	85	160	0
CF1	MOHI1	7/2/2024	2024	2024B	MT	P10	0.07	0.22	3	65	160	0
CF1	MOHI1	7/2/2024	2024	2024B	UT	P11	0.22	0.12	4	45	130.5	0
CF1	MOHI1	7/2/2024	2024	2024B	UT	P12	0.36	0.3	4	55	130.5	0
CF1	MOHI1	7/2/2024	2024	2024B	UT	P13	0.49	0.69	3	20	17	0
CF1	MOHI1	7/2/2024	2024	2024B	UT	P14	0.27	0.62	4	70	210	0
CF1	MOHI1	7/2/2024	2024	2024B	UT	P15	0.08	0.01	3	80	160	0
CF1	MOHI1	7/2/2024	2024	2024B	SS	P16	0.22	0.46	3	85	260	0
CF1	MOHI1	7/2/2024	2024	2024B	SS	P17	0.2	0.3	4	85	260	0

SiteN	WS	Date	Year	Ycat	Transect	Plot	Wdep	Wvel	SubRich	perBOCO	GSScore	ocrLWD
CF1	MOHI1	7/2/2024	2024	2024B	SS	P18	0.22	0.14	4	80	160	0
CF1	MOHI1	7/2/2024	2024	2024B	SS	P19	0.45	1.15	2	60	160	0
CF1	MOHI1	7/2/2024	2024	2024B	SS	P20	0.18	0.41	4	80	260	0
KR2	KOKO1	7/9/2024	2024	2024B	DT	P1	0.06	0.24	3	50	160	0
KR2	KOKO1	7/9/2024	2024	2024B	DT	P2	0.06	0.18	3	70	160	0
KR2	KOKO1	7/9/2024	2024	2024B	DT	P3	0.06	0.53	3	60	160	0
KR2	KOKO1	7/9/2024	2024	2024B	DT	P4	0.18	0.26	3	52	160	0
KR2	KOKO1	7/9/2024	2024	2024B	DT	P5	0.56	0.84	4	85	160	0
KR2	KOKO1	7/9/2024	2024	2024B	MT	P6	0.07	0.31	3	70	160	0
KR2	KOKO1	7/9/2024	2024	2024B	MT	P7	0.12	0.28	4	75	160	0
KR2	KOKO1	7/9/2024	2024	2024B	MT	P8	0.15	0.48	2	70	160	0
KR2	KOKO1	7/9/2024	2024	2024B	MT	P9	0.08	0.5	3	70	160	0
KR2	KOKO1	7/9/2024	2024	2024B	MT	P10	0.45	0.79	3	60	160	0
KR2	KOKO1	7/9/2024	2024	2024B	UT	P11	0.12	0.39	3	65	160	0
KR2	KOKO1	7/9/2024	2024	2024B	UT	P12	0.13	0.47	2	90	160	0
KR2	KOKO1	7/9/2024	2024	2024B	UT	P13	0.33	0.72	2	80	160	0
KR2	KOKO1	7/9/2024	2024	2024B	UT	P14	0.19	0.78	2	85	160	0
KR2	KOKO1	7/9/2024	2024	2024B	UT	P15	0.18	0.58	3	85	160	0
KR2	KOKO1	7/9/2024	2024	2024B	SS	P16	0.1	0.39	4	78	160	0
KR2	KOKO1	7/9/2024	2024	2024B	SS	P17	0.15	0.44	4	75	160	0
KR2	KOKO1	7/9/2024	2024	2024B	SS	P18	0.3	0.77	3	80	160	0
KR2	KOKO1	7/9/2024	2024	2024B	SS	P19	0.16	0.68	4	66	160	0
KR2	KOKO1	7/9/2024	2024	2024B	SS	P20	0.14	0.19	2	90	160	0
**Part 3 - Plot level riparian habitat and spatial variables**												
**SiteN**	**WS**	**Date**	**Year**	**Ycat**	**Transect**	**Plot**	**perCC**	**CIscore**	**dCHPm**	**dHPCm**		
KR1	KOKO1	6/5/2023	2023	2023A	DT	P1	0	0.33	1.53	6.83		
KR1	KOKO1	6/5/2023	2023	2023A	DT	P2	0	0.5	11.84	5.93		
KR1	KOKO1	6/5/2023	2023	2023A	DT	P3	30	0.67	1.4	5.35		
KR1	KOKO1	6/5/2023	2023	2023A	DT	P4	20	0.5	2.9	5.11		
KR1	KOKO1	6/5/2023	2023	2023A	DT	P5	60	0.33	4.3	5.27		

SiteN	WS	Date	Year	Ycat	Transect	Plot	perCC	CIscore	dCHPm	dHPCm		
KR1	KOKO1	6/5/2023	2023	2023A	MT	P1	60	0.67	7.2	6.61		
KR1	KOKO1	6/5/2023	2023	2023A	MT	P2	100	0.83	5.4	5.64		
KR1	KOKO1	6/5/2023	2023	2023A	MT	P3	100	1	3.6	5.14		
KR1	KOKO1	6/5/2023	2023	2023A	MT	P4	100	0.83	1.8	5.24		
KR1	KOKO1	6/5/2023	2023	2023A	MT	P5	95	0.67	11.84	5.92		
KR1	KOKO1	6/5/2023	2023	2023A	UT	P1	0	0.33	12.24	15.77		
KR1	KOKO1	6/5/2023	2023	2023A	UT	P2	0	0.5	11.15	15.39		
KR1	KOKO1	6/5/2023	2023	2023A	UT	P3	10	0.67	10.45	15.34		
KR1	KOKO1	6/5/2023	2023	2023A	UT	P4	60	0.5	10.22	15.63		
KR1	KOKO1	6/5/2023	2023	2023A	UT	P5	100	0.33	10.49	16.23		
KR1	KOKO1	6/5/2023	2023	2023A	SS	SS1	70	0.5	2.2	3.91		
KR1	KOKO1	6/5/2023	2023	2023A	SS	SS2	95	0.67	3.43	2.58		
KR1	KOKO1	6/5/2023	2023	2023A	SS	SS3	100	0.67	5.08	1.39		
KR1	KOKO1	6/5/2023	2023	2023A	SS	SS4	0	0.5	8.4	12.2		
KR1	KOKO1	6/5/2023	2023	2023A	SS	SS5	5	0.5	9.75	14.17		
KR3	KOKO1	6/6/2023	2023	2023A	DT	P1	80	0.33	20.05	37.27		
KR3	KOKO1	6/6/2023	2023	2023A	DT	P2	0	0.5	13.85	34.32		
KR3	KOKO1	6/6/2023	2023	2023A	DT	P3	0	0.67	8.57	32.53		
KR3	KOKO1	6/6/2023	2023	2023A	DT	P4	0	0.5	6.86	32.1		
KR3	KOKO1	6/6/2023	2023	2023A	DT	P5	60	0.33	10.59	33.07		
KR3	KOKO1	6/6/2023	2023	2023A	MT	P1	10	0.67	14.22	19.07		
KR3	KOKO1	6/6/2023	2023	2023A	MT	P2	0	0.83	7.11	12.2		
KR3	KOKO1	6/6/2023	2023	2023A	MT	P3	0	1	7.11	5.94		
KR3	KOKO1	6/6/2023	2023	2023A	MT	P4	0	0.83	7.11	4.78		
KR3	KOKO1	6/6/2023	2023	2023A	MT	P5	0	0.67	7.11	10.56		
KR3	KOKO1	6/6/2023	2023	2023A	UT	P1	10	0.33	15.72	31.05		
KR3	KOKO1	6/6/2023	2023	2023A	UT	P2	0	0.5	11.64	27.55		
KR3	KOKO1	6/6/2023	2023	2023A	UT	P3	0	0.67	10.11	25.15		
KR3	KOKO1	6/6/2023	2023	2023A	UT	P4	0	0.5	11.22	24.16		
KR3	KOKO1	6/6/2023	2023	2023A	UT	P5	0	0.33	6.24	24.77		
KR3	KOKO1	6/6/2023	2023	2023A	SS	SS1	0	0.5	21.58	25.36		

SiteN	WS	Date	Year	Ycat	Transect	Plot	perCC	CIscore	dCHPm	dHPCm		
KR3	KOKO1	6/6/2023	2023	2023A	SS	SS2	0	0.67	8.84	13.51		
KR3	KOKO1	6/6/2023	2023	2023A	SS	SS3	0	1	4.66	9.45		
KR3	KOKO1	6/6/2023	2023	2023A	SS	SS4	0	0.67	17.57	15.91		
KR3	KOKO1	6/6/2023	2023	2023A	SS	SS5	0	0.33	17.57	21.37		
AC1	ALUM1	6/29/2023	2023	2023A	DT	P1	10	0.33	10.78	11.35		
AC1	ALUM1	6/29/2023	2023	2023A	DT	P2	25	0.5	9.72	10.42		
AC1	ALUM1	6/29/2023	2023	2023A	DT	P3	25	0.67	9	9.84		
AC1	ALUM1	6/29/2023	2023	2023A	DT	P4	55	0.5	8.7	9.65		
AC1	ALUM1	6/29/2023	2023	2023A	DT	P5	55	0.33	8.88	9.89		
AC1	ALUM1	6/29/2023	2023	2023A	MT	P1	5	0.67	5.82	5.98		
AC1	ALUM1	6/29/2023	2023	2023A	MT	P2	35	0.83	3.89	3.92		
AC1	ALUM1	6/29/2023	2023	2023A	MT	P3	80	1	2.15	1.99		
AC1	ALUM1	6/29/2023	2023	2023A	MT	P4	90	0.83	2.06	1.03		
AC1	ALUM1	6/29/2023	2023	2023A	MT	P5	95	0.67	2	2.58		
AC1	ALUM1	6/29/2023	2023	2023A	UT	P1	0	0.33	8.71	9.7		
AC1	ALUM1	6/29/2023	2023	2023A	UT	P2	0	0.5	7.55	8.58		
AC1	ALUM1	6/29/2023	2023	2023A	UT	P3	10	0.67	6.89	7.89		
AC1	ALUM1	6/29/2023	2023	2023A	UT	P4	40	0.5	6.88	7.78		
AC1	ALUM1	6/29/2023	2023	2023A	UT	P5	75	0.33	7.52	8.25		
AC1	ALUM1	6/29/2023	2023	2023A	SS	SS1	45	0.83	4.56	5.41		
AC1	ALUM1	6/29/2023	2023	2023A	SS	SS2	60	0.67	2.26	3.15		
AC1	ALUM1	6/29/2023	2023	2023A	SS	SS3	75	0.83	1.27	2.26		
AC1	ALUM1	6/29/2023	2023	2023A	SS	SS4	95	0.83	2.06	1.03		
AC1	ALUM1	6/29/2023	2023	2023A	SS	SS5	80	0.83	1.79	2.82		
AC2	ALUM1	6/29/2023	2023	2023A	DT	P1	55	0.33	12.24	13.03		
AC2	ALUM1	6/29/2023	2023	2023A	DT	P2	35	0.5	12.1	12.73		
AC2	ALUM1	6/29/2023	2023	2023A	DT	P3	5	0.67	12.2	12.65		
AC2	ALUM1	6/29/2023	2023	2023A	DT	P4	0	0.5	12.53	12.8		
AC2	ALUM1	6/29/2023	2023	2023A	DT	P5	0	0.33	13.07	13.17		
AC2	ALUM1	6/29/2023	2023	2023A	MT	P1	90	0.67	2.32	3.35		
AC2	ALUM1	6/29/2023	2023	2023A	MT	P2	70	0.83	1.22	1.86		

SiteN	WS	Date	Year	Ycat	Transect	Plot	perCC	CIscore	dCHPm	dHPCm		
AC2	ALUM1	6/29/2023	2023	2023A	MT	P3	65	1	1.48	0.61		
AC2	ALUM1	6/29/2023	2023	2023A	MT	P4	30	0.83	2.74	1.37		
AC2	ALUM1	6/29/2023	2023	2023A	MT	P5	50	0.67	1.52	2.82		
AC2	ALUM1	6/29/2023	2023	2023A	UT	P1	25	0.33	13.52	13.85		
AC2	ALUM1	6/29/2023	2023	2023A	UT	P2	0	0.5	13.2	13.83		
AC2	ALUM1	6/29/2023	2023	2023A	UT	P3	0	0.67	13.63	14.52		
AC2	ALUM1	6/29/2023	2023	2023A	UT	P4	15	0.5	14.74	15.82		
AC2	ALUM1	6/29/2023	2023	2023A	UT	P5	100	0.33	16.4	17.6		
AC2	ALUM1	6/29/2023	2023	2023A	SS	SS1	80	0.67	3.3	3.05		
AC2	ALUM1	6/29/2023	2023	2023A	SS	SS2	80	0.83	2.74	1.37		
AC2	ALUM1	6/29/2023	2023	2023A	SS	SS3	30	0.83	3.73	4.16		
AC2	ALUM1	6/29/2023	2023	2023A	SS	SS4	65	0.67	4.87	4.73		
AC2	ALUM1	6/29/2023	2023	2023A	SS	SS5	80	0.67	7.36	7.28		
CF3	MOHI1	7/7/2023	2023	2023A	DT	P1	98	0.33	3.22	21.1		
CF3	MOHI1	7/7/2023	2023	2023A	DT	P2	98	0.5	3.21	20.57		
CF3	MOHI1	7/7/2023	2023	2023A	DT	P3	98	0.67	3.21	20.52		
CF3	MOHI1	7/7/2023	2023	2023A	DT	P4	0	0.5	3.25	20.99		
CF3	MOHI1	7/7/2023	2023	2023A	DT	P5	1	0.33	6.47	21.92		
CF3	MOHI1	7/7/2023	2023	2023A	MT	P1	40	0.67	3.33	6.67		
CF3	MOHI1	7/7/2023	2023	2023A	MT	P2	0	0.83	3.33	4.73		
CF3	MOHI1	7/7/2023	2023	2023A	MT	P3	0	1	3.33	4.74		
CF3	MOHI1	7/7/2023	2023	2023A	MT	P4	0	0.83	3.33	6.7		
CF3	MOHI1	7/7/2023	2023	2023A	MT	P5	20	0.67	3.33	9.45		
CF3	MOHI1	7/7/2023	2023	2023A	UT	P1	95	0.33	1.81	12.65		
CF3	MOHI1	7/7/2023	2023	2023A	UT	P2	95	0.5	1.5	11.74		
CF3	MOHI1	7/7/2023	2023	2023A	UT	P3	60	0.67	3.31	11.74		
CF3	MOHI1	7/7/2023	2023	2023A	UT	P4	0	0.5	3.31	12.63		
CF3	MOHI1	7/7/2023	2023	2023A	UT	P5	0	0.33	3.31	14.26		
CF3	MOHI1	7/7/2023	2023	2023A	SS	SS1	5	0.83	7.29	11.53		
CF3	MOHI1	7/7/2023	2023	2023A	SS	SS2	5	0.33	1.86	9.75		
CF3	MOHI1	7/7/2023	2023	2023A	SS	SS3	0	0.5	4.53	9.95		

SiteN	WS	Date	Year	Ycat	Transect	Plot	perCC	CIscore	dCHPm	dHPCm		
CF3	MOHI1	7/7/2023	2023	2023A	SS	SS4	95	0.33	1.81	14.2		
CF3	MOHI1	7/7/2023	2023	2023A	SS	SS5	85	0.5	1.5	12.93		
CF2	MOHI1	7/8/2023	2023	2023A	DT	P1	85	0.33	2.71	38.25		
CF2	MOHI1	7/8/2023	2023	2023A	DT	P2	20	0.5	11.25	37.97		
CF2	MOHI1	7/8/2023	2023	2023A	DT	P3	3	0.67	2.53	37.88		
CF2	MOHI1	7/8/2023	2023	2023A	DT	P4	0	0.5	5.14	37.97		
CF2	MOHI1	7/8/2023	2023	2023A	DT	P5	0	0.33	7.75	38.23		
CF2	MOHI1	7/8/2023	2023	2023A	MT	P1	75	0.67	5.17	5.57		
CF2	MOHI1	7/8/2023	2023	2023A	MT	P2	90	0.83	2.58	3.21		
CF2	MOHI1	7/8/2023	2023	2023A	MT	P3	90	1	2.58	1.69		
CF2	MOHI1	7/8/2023	2023	2023A	MT	P4	90	0.83	2.58	2.96		
CF2	MOHI1	7/8/2023	2023	2023A	MT	P5	80	0.67	2.58	5.29		
CF2	MOHI1	7/8/2023	2023	2023A	UT	P1	10	0.33	3.6	34.79		
CF2	MOHI1	7/8/2023	2023	2023A	UT	P2	10	0.5	7	34.53		
CF2	MOHI1	7/8/2023	2023	2023A	UT	P3	3	0.67	3.38	34.64		
CF2	MOHI1	7/8/2023	2023	2023A	UT	P4	0	0.5	7	35.09		
CF2	MOHI1	7/8/2023	2023	2023A	UT	P5	0	0.33	3.6	35.91		
CF2	MOHI1	7/8/2023	2023	2023A	SS	SS1	0	0.67	9.8	26.72		
CF2	MOHI1	7/8/2023	2023	2023A	SS	SS2	60	0.67	9.8	17.97		
CF2	MOHI1	7/8/2023	2023	2023A	SS	SS3	95	0.83	5.73	6.38		
CF2	MOHI1	7/8/2023	2023	2023A	SS	SS4	90	0.83	5.73	12.02		
CF2	MOHI1	7/8/2023	2023	2023A	SS	SS5	95	0.67	8.12	18.08		
BW2	UBWC1	7/11/2023	2023	2023A	DT	P1	25	0.33	NA	NA		
BW2	UBWC1	7/11/2023	2023	2023A	DT	P2	5	0.5	NA	NA		
BW2	UBWC1	7/11/2023	2023	2023A	DT	P3	0	0.67	NA	NA		
BW2	UBWC1	7/11/2023	2023	2023A	DT	P4	5	0.5	NA	NA		
BW2	UBWC1	7/11/2023	2023	2023A	DT	P5	20	0.33	NA	NA		
BW2	UBWC1	7/11/2023	2023	2023A	MT	P1	90	0.67	NA	NA		
BW2	UBWC1	7/11/2023	2023	2023A	MT	P2	10	0.83	NA	NA		
BW2	UBWC1	7/11/2023	2023	2023A	MT	P3	5	1	NA	NA		
BW2	UBWC1	7/11/2023	2023	2023A	MT	P4	0	0.83	NA	NA		

SiteN	WS	Date	Year	Ycat	Transect	Plot	perCC	CIscore	dCHPm	dHPCm		
BW2	UBWC1	7/11/2023	2023	2023A	MT	P5	5	0.67	NA	NA		
BW2	UBWC1	7/11/2023	2023	2023A	UT	P1	98	0.33	NA	NA		
BW2	UBWC1	7/11/2023	2023	2023A	UT	P2	20	0.5	NA	NA		
BW2	UBWC1	7/11/2023	2023	2023A	UT	P3	5	0.67	NA	NA		
BW2	UBWC1	7/11/2023	2023	2023A	UT	P4	0	0.5	NA	NA		
BW2	UBWC1	7/11/2023	2023	2023A	UT	P5	1	0.33	NA	NA		
BW2	UBWC1	7/11/2023	2023	2023A	SS	SS1	0	0.67	NA	NA		
BW2	UBWC1	7/11/2023	2023	2023A	SS	SS2	0	0.67	NA	NA		
BW2	UBWC1	7/11/2023	2023	2023A	SS	SS3	5	0.83	NA	NA		
BW2	UBWC1	7/11/2023	2023	2023A	SS	SS4	0	0.83	NA	NA		
BW2	UBWC1	7/11/2023	2023	2023A	SS	SS5	0	0.67	NA	NA		
BW1	UBWC1	7/11/2023	2023	2023A	DT	P1	99	0.33	NA	NA		
BW1	UBWC1	7/11/2023	2023	2023A	DT	P2	60	0.5	NA	NA		
BW1	UBWC1	7/11/2023	2023	2023A	DT	P3	0	0.67	NA	NA		
BW1	UBWC1	7/11/2023	2023	2023A	DT	P4	0	0.5	NA	NA		
BW1	UBWC1	7/11/2023	2023	2023A	DT	P5	0	0.33	NA	NA		
BW1	UBWC1	7/11/2023	2023	2023A	MT	P1	99	0.67	NA	NA		
BW1	UBWC1	7/11/2023	2023	2023A	MT	P2	99	0.83	NA	NA		
BW1	UBWC1	7/11/2023	2023	2023A	MT	P3	45	1	NA	NA		
BW1	UBWC1	7/11/2023	2023	2023A	MT	P4	0	0.83	NA	NA		
BW1	UBWC1	7/11/2023	2023	2023A	MT	P5	0	0.67	NA	NA		
BW1	UBWC1	7/11/2023	2023	2023A	UT	P1	5	0.33	NA	NA		
BW1	UBWC1	7/11/2023	2023	2023A	UT	P2	0	0.5	NA	NA		
BW1	UBWC1	7/11/2023	2023	2023A	UT	P3	0	0.67	NA	NA		
BW1	UBWC1	7/11/2023	2023	2023A	UT	P4	0	0.5	NA	NA		
BW1	UBWC1	7/11/2023	2023	2023A	UT	P5	0	0.33	NA	NA		
BW1	UBWC1	7/11/2023	2023	2023A	SS	SS1	0	0.33	NA	NA		
BW1	UBWC1	7/11/2023	2023	2023A	SS	SS2	95	0.67	NA	NA		
BW1	UBWC1	7/11/2023	2023	2023A	SS	SS3	99	0.33	NA	NA		
BW1	UBWC1	7/11/2023	2023	2023A	SS	SS4	0	0.5	NA	NA		
BW1	UBWC1	7/11/2023	2023	2023A	SS	SS5	0	0.67	NA	NA		

SiteN	WS	Date	Year	Ycat	Transect	Plot	perCC	CIscore	dCHPm	dHPCm		
CF1	MOHI1	7/19/2023	2023	2023A	DT	P1	35	0.33	6.66	15.24		
CF1	MOHI1	7/19/2023	2023	2023A	DT	P2	0	0.5	3.33	13.98		
CF1	MOHI1	7/19/2023	2023	2023A	DT	P3	0	0.67	3.33	13.45		
CF1	MOHI1	7/19/2023	2023	2023A	DT	P4	0	0.5	3.33	13.73		
CF1	MOHI1	7/19/2023	2023	2023A	DT	P5	0	0.33	3.33	14.78		
CF1	MOHI1	7/19/2023	2023	2023A	MT	P1	0	0.67	3.83	6.68		
CF1	MOHI1	7/19/2023	2023	2023A	MT	P2	0	0.83	3.83	2.85		
CF1	MOHI1	7/19/2023	2023	2023A	MT	P3	0	1	2.21	0.99		
CF1	MOHI1	7/19/2023	2023	2023A	MT	P4	0	0.83	3.83	4.82		
CF1	MOHI1	7/19/2023	2023	2023A	MT	P5	5	0.67	3.83	8.65		
CF1	MOHI1	7/19/2023	2023	2023A	UT	P1	0	0.33	3.42	15.04		
CF1	MOHI1	7/19/2023	2023	2023A	UT	P2	0	0.5	3.41	13.76		
CF1	MOHI1	7/19/2023	2023	2023A	UT	P3	0	0.67	3.41	13.26		
CF1	MOHI1	7/19/2023	2023	2023A	UT	P4	1	0.5	3.42	13.63		
CF1	MOHI1	7/19/2023	2023	2023A	UT	P5	10	0.33	3.42	14.8		
CF1	MOHI1	7/19/2023	2023	2023A	SS	SS1	0	0.5	3.96	10.19		
CF1	MOHI1	7/19/2023	2023	2023A	SS	SS2	0	0.67	3.28	8.85		
CF1	MOHI1	7/19/2023	2023	2023A	SS	SS3	0	0.83	3.28	5.74		
CF1	MOHI1	7/19/2023	2023	2023A	SS	SS4	0	1	2.21	2.27		
CF1	MOHI1	7/19/2023	2023	2023A	SS	SS5	0	0.67	3.67	9.61		
KR1	KOKO1	6/4/2024	2024	2024B	DT	P1	3	0.33	5.72	9.86		
KR1	KOKO1	6/4/2024	2024	2024B	DT	P2	35	0.5	4.57	9.33		
KR1	KOKO1	6/4/2024	2024	2024B	DT	P3	70	0.67	3.55	8.97		
KR1	KOKO1	6/4/2024	2024	2024B	DT	P4	95	0.5	2.79	8.79		
KR1	KOKO1	6/4/2024	2024	2024B	DT	P5	99	0.33	2.55	8.8		
KR1	KOKO1	6/4/2024	2024	2024B	MT	P6	15	0.67	6.32	4.24		
KR1	KOKO1	6/4/2024	2024	2024B	MT	P7	90	0.83	5.43	2.58		
KR1	KOKO1	6/4/2024	2024	2024B	MT	P8	100	1	5.12	1.63		
KR1	KOKO1	6/4/2024	2024	2024B	MT	P9	100	0.83	5.5	2.44		
KR1	KOKO1	6/4/2024	2024	2024B	MT	P10	95	0.67	6.45	4.07		
KR1	KOKO1	6/4/2024	2024	2024B	UT	P11	0	0.33	6.02	12.42		

SiteN	WS	Date	Year	Ycat	Transect	Plot	perCC	CIscore	dCHPm	dHPCm		
KR1	KOKO1	6/4/2024	2024	2024B	UT	P12	0	0.5	5.28	12.04		
KR1	KOKO1	6/4/2024	2024	2024B	UT	P13	20	0.67	5.87	12.26		
KR1	KOKO1	6/4/2024	2024	2024B	UT	P14	40	0.5	7.48	13.07		
KR1	KOKO1	6/4/2024	2024	2024B	UT	P15	80	0.33	9.61	14.35		
KR1	KOKO1	6/4/2024	2024	2024B	SS	P16	60	0.67	4.29	9.93		
KR1	KOKO1	6/4/2024	2024	2024B	SS	P17	95	0.5	2.19	7.83		
KR1	KOKO1	6/4/2024	2024	2024B	SS	P18	99	0.5	1.28	7.48		
KR1	KOKO1	6/4/2024	2024	2024B	SS	P19	99	0.33	13.01	6.26		
KR1	KOKO1	6/4/2024	2024	2024B	SS	P20	20	0.83	13.01	6.75		
KR3	KOKO1	6/25/2024	2024	2024B	DT	P1	0	0.33	23.88	32.3		
KR3	KOKO1	6/25/2024	2024	2024B	DT	P2	0	0.5	17.62	29.85		
KR3	KOKO1	6/25/2024	2024	2024B	DT	P3	0	0.67	11.66	28.71		
KR3	KOKO1	6/25/2024	2024	2024B	DT	P4	0	0.5	6.82	29.03		
KR3	KOKO1	6/25/2024	2024	2024B	DT	P5	0	0.33	6.51	30.77		
KR3	KOKO1	6/25/2024	2024	2024B	MT	P6	0	0.67	11.38	14.38		
KR3	KOKO1	6/25/2024	2024	2024B	MT	P7	0	0.83	7.23	7.32		
KR3	KOKO1	6/25/2024	2024	2024B	MT	P8	0	1	8.68	0.81		
KR3	KOKO1	6/25/2024	2024	2024B	MT	P9	0	0.83	14.09	6.92		
KR3	KOKO1	6/25/2024	2024	2024B	MT	P10	0	0.67	20.55	13.98		
KR3	KOKO1	6/25/2024	2024	2024B	UT	P11	5	0.33	20.85	32.77		
KR3	KOKO1	6/25/2024	2024	2024B	UT	P12	0	0.5	14.81	30.36		
KR3	KOKO1	6/25/2024	2024	2024B	UT	P13	0	0.67	9.48	29.25		
KR3	KOKO1	6/25/2024	2024	2024B	UT	P14	0	0.5	6.81	29.59		
KR3	KOKO1	6/25/2024	2024	2024B	UT	P15	0	0.33	9.43	31.33		
KR3	KOKO1	6/25/2024	2024	2024B	SS	P16	0	0.5	33.5	24.3		
KR3	KOKO1	6/25/2024	2024	2024B	SS	P17	0	0.67	13.7	12.26		
KR3	KOKO1	6/25/2024	2024	2024B	SS	P18	0	1	15.23	5.8		
KR3	KOKO1	6/25/2024	2024	2024B	SS	P19	0	0.67	17.84	9.43		
KR3	KOKO1	6/25/2024	2024	2024B	SS	P20	0	0.5	17.84	22.91		
AC1	ALUM1	6/10/2024	2024	2024B	DT	P1	35	0.33	10.88	10.05		
AC1	ALUM1	6/10/2024	2024	2024B	DT	P2	45	0.5	9.8	9.26		

SiteN	WS	Date	Year	Ycat	Transect	Plot	perCC	CIscore	dCHPm	dHPCm		
AC1	ALUM1	6/10/2024	2024	2024B	DT	P3	65	0.67	9.02	8.84		
AC1	ALUM1	6/10/2024	2024	2024B	DT	P4	50	0.5	8.63	8.85		
AC1	ALUM1	6/10/2024	2024	2024B	DT	P5	50	0.33	8.69	9.3		
AC1	ALUM1	6/10/2024	2024	2024B	MT	P6	0	0.67	6.48	4.69		
AC1	ALUM1	6/10/2024	2024	2024B	MT	P7	40	0.83	4.32	2.54		
AC1	ALUM1	6/10/2024	2024	2024B	MT	P8	80	1	2.16	0.42		
AC1	ALUM1	6/10/2024	2024	2024B	MT	P9	95	0.83	NA	1.8		
AC1	ALUM1	6/10/2024	2024	2024B	MT	P10	99	0.67	2.16	3.95		
AC1	ALUM1	6/10/2024	2024	2024B	UT	P11	0	0.33	9.95	8.74		
AC1	ALUM1	6/10/2024	2024	2024B	UT	P12	0	0.5	8.68	7.79		
AC1	ALUM1	6/10/2024	2024	2024B	UT	P13	10	0.67	7.85	7.41		
AC1	ALUM1	6/10/2024	2024	2024B	UT	P14	60	0.5	7.6	7.68		
AC1	ALUM1	6/10/2024	2024	2024B	UT	P15	85	0.33	7.99	8.54		
AC1	ALUM1	6/10/2024	2024	2024B	SS	P16	85	1	3.09	1.98		
AC1	ALUM1	6/10/2024	2024	2024B	SS	P17	95	0.83	0.53	1.44		
AC1	ALUM1	6/10/2024	2024	2024B	SS	P18	85	0.67	3.36	3.32		
AC1	ALUM1	6/10/2024	2024	2024B	SS	P19	95	0.67	3.65	4.57		
AC1	ALUM1	6/10/2024	2024	2024B	SS	P20	90	0.67	4.14	4.56		
AC2	ALUM1	6/11/2024	2024	2024B	DT	P1	75	0.33	NA	NA		
AC2	ALUM1	6/11/2024	2024	2024B	DT	P2	30	0.5	NA	NA		
AC2	ALUM1	6/11/2024	2024	2024B	DT	P3	20	0.67	NA	NA		
AC2	ALUM1	6/11/2024	2024	2024B	DT	P4	10	0.5	NA	NA		
AC2	ALUM1	6/11/2024	2024	2024B	DT	P5	0	0.33	NA	NA		
AC2	ALUM1	6/11/2024	2024	2024B	MT	P6	95	0.67	NA	NA		
AC2	ALUM1	6/11/2024	2024	2024B	MT	P7	85	0.83	NA	NA		
AC2	ALUM1	6/11/2024	2024	2024B	MT	P8	50	1	NA	NA		
AC2	ALUM1	6/11/2024	2024	2024B	MT	P9	50	0.83	NA	NA		
AC2	ALUM1	6/11/2024	2024	2024B	MT	P10	75	0.67	NA	NA		
AC2	ALUM1	6/11/2024	2024	2024B	UT	P11	2	0.33	NA	NA		
AC2	ALUM1	6/11/2024	2024	2024B	UT	P12	0	0.5	NA	NA		
AC2	ALUM1	6/11/2024	2024	2024B	UT	P13	2	0.67	NA	NA		

SiteN	WS	Date	Year	Ycat	Transect	Plot	perCC	CIscore	dCHPm	dHPCm		
AC2	ALUM1	6/11/2024	2024	2024B	UT	P14	35	0.5	NA	NA		
AC2	ALUM1	6/11/2024	2024	2024B	UT	P15	85	0.33	NA	NA		
AC2	ALUM1	6/11/2024	2024	2024B	SS	P16	75	0.67	NA	NA		
AC2	ALUM1	6/11/2024	2024	2024B	SS	P17	55	0.67	NA	NA		
AC2	ALUM1	6/11/2024	2024	2024B	SS	P18	80	1	NA	NA		
AC2	ALUM1	6/11/2024	2024	2024B	SS	P19	99	0.5	NA	NA		
AC2	ALUM1	6/11/2024	2024	2024B	SS	P20	85	0.5	NA	NA		
CF3	MOHI1	6/17/2024	2024	2024B	DT	P1	98	0.33	2.82	17.42		
CF3	MOHI1	6/17/2024	2024	2024B	DT	P2	95	0.5	3.12	16.73		
CF3	MOHI1	6/17/2024	2024	2024B	DT	P3	90	0.67	3.12	16.6		
CF3	MOHI1	6/17/2024	2024	2024B	DT	P4	0	0.5	3.12	17.06		
CF3	MOHI1	6/17/2024	2024	2024B	DT	P5	5	0.33	6.24	18.06		
CF3	MOHI1	6/17/2024	2024	2024B	MT	P6	60	0.67	3.3	5.21		
CF3	MOHI1	6/17/2024	2024	2024B	MT	P7	5	0.83	3.3	2.04		
CF3	MOHI1	6/17/2024	2024	2024B	MT	P8	0	1	3.3	1.71		
CF3	MOHI1	6/17/2024	2024	2024B	MT	P9	0	0.83	3.3	4.85		
CF3	MOHI1	6/17/2024	2024	2024B	MT	P10	20	0.67	3.31	8.13		
CF3	MOHI1	6/17/2024	2024	2024B	UT	P11	100	0.33	2.85	18.15		
CF3	MOHI1	6/17/2024	2024	2024B	UT	P12	90	0.5	3.02	17.49		
CF3	MOHI1	6/17/2024	2024	2024B	UT	P13	95	0.67	3.02	17.33		
CF3	MOHI1	6/17/2024	2024	2024B	UT	P14	25	0.5	2.94	17.7		
CF3	MOHI1	6/17/2024	2024	2024B	UT	P15	5	0.33	3.02	18.55		
CF3	MOHI1	6/17/2024	2024	2024B	SS	P16	100	0.33	1.73	15.18		
CF3	MOHI1	6/17/2024	2024	2024B	SS	P17	99	0.5	1.73	14.29		
CF3	MOHI1	6/17/2024	2024	2024B	SS	P18	100	0.67	4.27	10.03		
CF3	MOHI1	6/17/2024	2024	2024B	SS	P19	95	0.33	2.85	15.45		
CF3	MOHI1	6/17/2024	2024	2024B	SS	P20	0	0.5	2.94	15.11		
CF2	MOHI1	7/1/2024	2024	2024B	DT	P1	15	0.33	2.19	31.69		
CF2	MOHI1	7/1/2024	2024	2024B	DT	P2	3	0.5	2.19	31.36		
CF2	MOHI1	7/1/2024	2024	2024B	DT	P3	0	0.67	2.19	31.18		
CF2	MOHI1	7/1/2024	2024	2024B	DT	P4	0	0.5	2.19	31.16		

SiteN	WS	Date	Year	Ycat	Transect	Plot	perCC	CIscore	dCHPm	dHPCm		
CF2	MOHI1	7/1/2024	2024	2024B	DT	P5	0	0.33	4.38	31.29		
CF2	MOHI1	7/1/2024	2024	2024B	MT	P6	90	0.67	4.14	5.82		
CF2	MOHI1	7/1/2024	2024	2024B	MT	P7	95	0.83	2.51	3.56		
CF2	MOHI1	7/1/2024	2024	2024B	MT	P8	95	1	2.51	2		
CF2	MOHI1	7/1/2024	2024	2024B	MT	P9	95	0.83	2.51	2.82		
CF2	MOHI1	7/1/2024	2024	2024B	MT	P10	90	0.67	2.51	4.95		
CF2	MOHI1	7/1/2024	2024	2024B	UT	P11	1	0.33	3.45	34.33		
CF2	MOHI1	7/1/2024	2024	2024B	UT	P12	3	0.33	3.45	34.05		
CF2	MOHI1	7/1/2024	2024	2024B	UT	P13	0	0.5	3.45	34.11		
CF2	MOHI1	7/1/2024	2024	2024B	UT	P14	0	0.5	3.45	34.52		
CF2	MOHI1	7/1/2024	2024	2024B	UT	P15	0	0.67	6.9	35.26		
CF2	MOHI1	7/1/2024	2024	2024B	SS	P16	95	0.67	5.42	19.78		
CF2	MOHI1	7/1/2024	2024	2024B	SS	P17	85	0.67	5.42	15.08		
CF2	MOHI1	7/1/2024	2024	2024B	SS	P18	90	0.83	4.43	3.16		
CF2	MOHI1	7/1/2024	2024	2024B	SS	P19	95	0.83	2.88	4.8		
CF2	MOHI1	7/1/2024	2024	2024B	SS	P20	60	0.83	5.69	8.65		
BW2	UBWC1	6/18/2024	2024	2024B	DT	P1	50	0.33	NA	NA		
BW2	UBWC1	6/18/2024	2024	2024B	DT	P2	35	0.5	NA	NA		
BW2	UBWC1	6/18/2024	2024	2024B	DT	P3	5	0.67	NA	NA		
BW2	UBWC1	6/18/2024	2024	2024B	DT	P4	0	0.5	NA	NA		
BW2	UBWC1	6/18/2024	2024	2024B	DT	P5	30	0.33	NA	NA		
BW2	UBWC1	6/18/2024	2024	2024B	MT	P6	20	0.67	NA	NA		
BW2	UBWC1	6/18/2024	2024	2024B	MT	P7	10	0.83	NA	NA		
BW2	UBWC1	6/18/2024	2024	2024B	MT	P8	2	1	NA	NA		
BW2	UBWC1	6/18/2024	2024	2024B	MT	P9	0	0.83	NA	NA		
BW2	UBWC1	6/18/2024	2024	2024B	MT	P10	10	0.67	NA	NA		
BW2	UBWC1	6/18/2024	2024	2024B	UT	P11	95	0.33	NA	NA		
BW2	UBWC1	6/18/2024	2024	2024B	UT	P12	50	0.5	NA	NA		
BW2	UBWC1	6/18/2024	2024	2024B	UT	P13	15	0.67	NA	NA		
BW2	UBWC1	6/18/2024	2024	2024B	UT	P14	5	0.5	NA	NA		
BW2	UBWC1	6/18/2024	2024	2024B	UT	P15	0	0.33	NA	NA		

SiteN	WS	Date	Year	Ycat	Transect	Plot	perCC	CIscore	dCHPm	dHPCm		
BW2	UBWC1	6/18/2024	2024	2024B	SS	P16	15	0.67	NA	NA		
BW2	UBWC1	6/18/2024	2024	2024B	SS	P17	5	0.83	NA	NA		
BW2	UBWC1	6/18/2024	2024	2024B	SS	P18	80	0.67	NA	NA		
BW2	UBWC1	6/18/2024	2024	2024B	SS	P19	0	0.67	NA	NA		
BW2	UBWC1	6/18/2024	2024	2024B	SS	P20	20	0.5	NA	NA		
BW1	UBWC1	6/24/2024	2024	2024B	DT	P1	100	0.33	31.77	16.75		
BW1	UBWC1	6/24/2024	2024	2024B	DT	P2	100	0.5	31.66	16.58		
BW1	UBWC1	6/24/2024	2024	2024B	DT	P3	30	0.67	31.65	16.59		
BW1	UBWC1	6/24/2024	2024	2024B	DT	P4	5	0.5	31.73	16.79		
BW1	UBWC1	6/24/2024	2024	2024B	DT	P5	15	0.33	31.91	17.18		
BW1	UBWC1	6/24/2024	2024	2024B	MT	P6	0	0.67	15.15	2.5		
BW1	UBWC1	6/24/2024	2024	2024B	MT	P7	0	0.83	14.91	0.72		
BW1	UBWC1	6/24/2024	2024	2024B	MT	P8	0	1	14.89	1.13		
BW1	UBWC1	6/24/2024	2024	2024B	MT	P9	0	0.83	15.08	2.92		
BW1	UBWC1	6/24/2024	2024	2024B	MT	P10	0	0.67	15.48	4.71		
BW1	UBWC1	6/24/2024	2024	2024B	UT	P11	10	0.33	3.72	16.3		
BW1	UBWC1	6/24/2024	2024	2024B	UT	P12	0	0.5	2.69	16.12		
BW1	UBWC1	6/24/2024	2024	2024B	UT	P13	0	0.67	1.72	16.02		
BW1	UBWC1	6/24/2024	2024	2024B	UT	P14	0	0.5	0.98	15.98		
BW1	UBWC1	6/24/2024	2024	2024B	UT	P15	0	0.33	1.12	16.02		
BW1	UBWC1	6/24/2024	2024	2024B	SS	P16	30	0.67	26.15	11.07		
BW1	UBWC1	6/24/2024	2024	2024B	SS	P17	35	0.5	25.72	10.69		
BW1	UBWC1	6/24/2024	2024	2024B	SS	P18	0	0.5	20.82	6.24		
BW1	UBWC1	6/24/2024	2024	2024B	SS	P19	0	0.67	1.87	13.97		
BW1	UBWC1	6/24/2024	2024	2024B	SS	P20	0	0.5	NA	15.09		
CF1	MOHI1	7/2/2024	2024	2024B	DT	P1	10	0.33	7.04	14.48		
CF1	MOHI1	7/2/2024	2024	2024B	DT	P2	0	0.5	3.52	13.08		
CF1	MOHI1	7/2/2024	2024	2024B	DT	P3	0	0.67	3.52	12.54		
CF1	MOHI1	7/2/2024	2024	2024B	DT	P4	0	0.5	3.52	12.97		
CF1	MOHI1	7/2/2024	2024	2024B	DT	P5	20	0.33	3.52	14.28		
CF1	MOHI1	7/2/2024	2024	2024B	MT	P6	0	0.67	3.73	7.03		

SiteN	WS	Date	Year	Ycat	Transect	Plot	perCC	CIscore	dCHPm	dHPCm		
CF1	MOHI1	7/2/2024	2024	2024B	MT	P7	0	0.83	1.28	3.3		
CF1	MOHI1	7/2/2024	2024	2024B	MT	P8	0	1	3.66	0.44		
CF1	MOHI1	7/2/2024	2024	2024B	MT	P9	0	0.83	3.73	4.16		
CF1	MOHI1	7/2/2024	2024	2024B	MT	P10	5	0.67	3.73	7.89		
CF1	MOHI1	7/2/2024	2024	2024B	UT	P11	30	0.33	7.94	13.58		
CF1	MOHI1	7/2/2024	2024	2024B	UT	P12	0	0.5	4.91	12.08		
CF1	MOHI1	7/2/2024	2024	2024B	UT	P13	5	0.67	2.67	11.39		
CF1	MOHI1	7/2/2024	2024	2024B	UT	P14	25	0.5	1.92	11.64		
CF1	MOHI1	7/2/2024	2024	2024B	UT	P15	35	0.33	1.81	12.77		
CF1	MOHI1	7/2/2024	2024	2024B	SS	P16	5	0.5	3.64	9.87		
CF1	MOHI1	7/2/2024	2024	2024B	SS	P17	0	0.67	6.15	7.38		
CF1	MOHI1	7/2/2024	2024	2024B	SS	P18	0	0.83	1.28	3.23		
CF1	MOHI1	7/2/2024	2024	2024B	SS	P19	0	0.67	5.35	8.84		
CF1	MOHI1	7/2/2024	2024	2024B	SS	P20	15	0.5	5.35	12.93		
KR2	KOKO1	7/9/2024	2024	2024B	DT	P1	0	0.33	19.06	18.17		
KR2	KOKO1	7/9/2024	2024	2024B	DT	P2	0	0.5	17.65	17.12		
KR2	KOKO1	7/9/2024	2024	2024B	DT	P3	0	0.67	16.63	16.51		
KR2	KOKO1	7/9/2024	2024	2024B	DT	P4	0	0.5	16.07	16.39		
KR2	KOKO1	7/9/2024	2024	2024B	DT	P5	0	0.33	16.01	16.77		
KR2	KOKO1	7/9/2024	2024	2024B	MT	P6	0	0.67	9.96	7.46		
KR2	KOKO1	7/9/2024	2024	2024B	MT	P7	0	0.83	6.64	4.15		
KR2	KOKO1	7/9/2024	2024	2024B	MT	P8	0	1	3.32	0.9		
KR2	KOKO1	7/9/2024	2024	2024B	MT	P9	5	0.83	NA	2.54		
KR2	KOKO1	7/9/2024	2024	2024B	MT	P10	15	0.67	3.32	5.84		
KR2	KOKO1	7/9/2024	2024	2024B	UT	P11	0	0.33	17.65	16.09		
KR2	KOKO1	7/9/2024	2024	2024B	UT	P12	0	0.5	15.92	14.85		
KR2	KOKO1	7/9/2024	2024	2024B	UT	P13	0	0.67	15.04	14.59		
KR2	KOKO1	7/9/2024	2024	2024B	UT	P14	0	0.5	15.15	15.37		
KR2	KOKO1	7/9/2024	2024	2024B	UT	P15	3	0.33	16.24	17.03		
KR2	KOKO1	7/9/2024	2024	2024B	SS	P16	0	0.67	9.02	7.76		
KR2	KOKO1	7/9/2024	2024	2024B	SS	P17	0	0.83	2.01	1.81		

SiteN	WS	Date	Year	Ycat	Transect	Plot	perCC	CIscore	dCHPm	dHPCm		
KR2	KOKO1	7/9/2024	2024	2024B	SS	P18	3	0.83	2.65	4.62		
KR2	KOKO1	7/9/2024	2024	2024B	SS	P19	0	0.83	3.07	2.77		
KR2	KOKO1	7/9/2024	2024	2024B	SS	P20	0	0.5	13.57	14.18		
**Part 4 - Latitude and longitude for each riffle (SiteN)**												
**SiteN**	**WS**	**Lat**	**Long**									
AC1	ALUM1	40.3833	82.8776									
AC2	ALUM1	40.3900	82.8744									
BW1	UBWC1	40.2183	82.8755									
BW2	UBWC1	40.2183	82.8755									
CF1	MOHI1	40.6131	82.3161									
CF2	MOHI1	40.6108	82.3072									
CF3	MOHI1	40.6078	82.2656									
KR1	KOKO1	40.4089	82.3251									
KR2	KOKO1	40.3730	82.4648									
KR3	KOKO1	40.3782	82.2386									
