# Supplementary material for: Small-Scale Habitat Relationships of Corydalus cornutus Hellgrammites in Central Ohio Riffles
Source: Insects. 2026 Apr 10;17(4):410. doi: 10.3390/insects17040410 (PMC13115823; doi:10.3390/insects17040410)
Supplement: Supplementary file 1 [file insects-17-00410-s001.zip › insects-4189354-supplementary/BossleyetalSupplementaryFile2-Insects.pdf]

Supplementary File S2. R code for Bossley et al. 2026. Small-scale habitat relationships of *Corydalus cornutus* hellgrammites in central Ohio riffles. *Insects.*

1. R code for mixed effects regression tree analyses of *Corydalus cornutus* hellgrammite occurrence, density, and mean head capsule width

```
///bring in data and check

P1 <- read.csv(file.choose(), header=TRUE)

head(P1)
tail(P1)
summary(P1)

attach(P1)


///load needed packages

require(glmertree)


///run glmertree with density (pDen)

tre1<-glmertree(pDen ~ 1|(1|WS/SiteN)| Wdep + Wvel + SubRich + perBOCO + GSScore + ocrLWD + perCC + CIscore + dCHPm + dHPCm, data = P1, family = "poisson", na.action = na.exclude)

plot(tre1)
print(tre1)
summary(tre1)
print(tre1$glmer)

AIC(tre1)


/run null model for density

treNg<-glmertree(pDen ~ 1 | (1|WS/SiteN)| 1, data = P1, family = "poisson", na.action = na.exclude)

plot(treNg)
print(treNg)
summary(treNg)
print(treNg$glmer)
AIC(treNg)


///run linear mixed effects regression tree with log(x+1) transformed head capsule width (lgHCW)
```

```
trHC1<-lmertree(lgHCW ~ 1|(1|WS/SiteN)| Wdep + Wvel + SubRich + perBOCO + GSScore +
ocrLWD + perCC + CIscore + dCHPm + dHPCm, data = P1, na.action = na.exclude)

plot(trHC1)
print(trHC1)
summary(trHC1)
print(trHC1$lmer)

AIC(trHC1)


/run null model for log(x+1) transformed head capsule width (lgHCW)

trHCN<-lmertree(lgHCW ~ 1|(1|WS/SiteN)| 1, data = P1, na.action = na.exclude)

print(trHCN)
summary(trHCN)
print(trHCN$lmer)

AIC(trHCN)


///run generalized linear mixed effects model regression tree analysis for occurrence (pOCR)

trOCR1 <-glmertree(pOCR ~ 1| (1|WS/SiteN) | Wdep + Wvel + SubRich + perBOCO + GSScore +
ocrLWD + perCC + CIscore + dCHPm + dHPCm, data = P1, family = "binomial", na.action =
na.exclude)

plot(trOCR1)
print(trOCR1)
summary(trOCR1)
print(trOCR1$glmer)

AIC(trOCR1)


/run null model for occurrence (pOCR)


trOCRN <-glmertree(pOCR ~ 1| (1|WS/SiteN) | 1, data = P1, family = "binomial", na.action =
na.exclude)

plot(trOCRN)
print(trOCRN)
summary(trOCRN)
print(trOCRN$glmer)

AIC(trOCRN)
```

2. R code for linear and generalized linear mixed effects model analyses of *Corydalus cornutus* hellgrammite occurrence, density, and mean head capsule width

///load needed packages

```
require(glmmTMB)
require(car)
```

///preliminary analyses to identify best random effects, model residual evaluations, and zero inflation terms

//evaluation of different random effects

/evaluation of random effects for density (pDen)

```
d1 <-glmmTMB(pDen ~ Wdep + perCC + CIscore + perBOCO + (1|SiteN), REML = TRUE, data =
P1, na.action = na.exclude)
d2 <-glmmTMB(pDen ~ Wdep + perCC + CIscore + perBOCO + (1|WS), REML = TRUE, data = P1,
na.action = na.exclude)
d3 <-glmmTMB(pDen ~ Wdep + perCC + CIscore + perBOCO + (1|WS/SiteN), REML = TRUE, data
= P1, na.action = na.exclude)
d4 <-glmmTMB(pDen ~ Wdep + perCC + CIscore + perBOCO + (1|WS) + (1|SiteN), REML = TRUE,
data = P1, na.action = na.exclude)
d5 <-glmmTMB(pDen ~ Wdep + perCC + CIscore + perBOCO + (1|Ycat), REML = TRUE, data = P1,
na.action = na.exclude)

AIC(d1, d2, d3, d4, d5)
```

/evaluation of random effects for mean head capsule width (pmnHCW)

```
d6 <-glmmTMB(pmnHCW ~ Wdep + perCC + CIscore + perBOCO + (1|SiteN), data = P1, REML =
TRUE, na.action = na.exclude)
d7 <-glmmTMB(pmnHCW ~ Wdep + perCC + CIscore + perBOCO + (1|WS), data = P1, REML =
TRUE, na.action = na.exclude)
d8 <-glmmTMB(pmnHCW ~ Wdep + perCC + CIscore + perBOCO + (1|WS/SiteN), data = P1,
REML = TRUE, na.action = na.exclude)
d9 <-glmmTMB(pmnHCW ~ Wdep + perCC + CIscore + perBOCO + (1|WS) + (1|SiteN), data = P1,
REML = TRUE, na.action = na.exclude)
d10 <-glmmTMB(pmnHCW ~ Wdep + perCC + CIscore + perBOCO + (1|Ycat), data = P1, REML =
TRUE, na.action = na.exclude)

AIC(d6, d7, d8, d9, d10)
```

/ evaluation of random effects for occurrence (pOCR)

```
d11 <-glmmTMB(pOCR ~ Wdep + perCC + CIscore + perBOCO + (1|SiteN), family = binomial, data
= P1, na.action = na.exclude)
d12 <-glmmTMB(pOCR ~ Wdep + perCC + CIscore + perBOCO + (1|WS), family = binomial, data =
P1, na.action = na.exclude)
d13 <-glmmTMB(pOCR ~ Wdep + perCC + CIscore + perBOCO + (1|WS/SiteN), family = binomial,
data = P1, na.action = na.exclude)
d14 <-glmmTMB(pOCR ~ Wdep + perCC + CIscore + perBOCO + (1|WS) + (1|SiteN), family =
binomial, data = P1, na.action = na.exclude)
d15 <-glmmTMB(pOCR ~ Wdep + perCC + CIscore + perBOCO + (1|Ycat), family = binomial, data
= P1, na.action = na.exclude)

AIC(d11, d12, d13, d14, d15)


//evaluation of model residuals with random effect of sites nested within watersheds (WS/SiteN)

/model residual evaluation for density (pDen)

d3 <-glmmTMB(pDen ~ Wdep + perCC + CIscore + perBOCO + (1|WS/SiteN), REML = FALSE,
data = P1, na.action = na.exclude)

res.d3<-residuals(d3)
qqPlot(res.d3)
shapiro.test(res.d3)


fit.d3<-fitted(d3)
plot(res.d3 ~ fit.d3)

/ model residual evaluation for mean head capsule width (pmnHCW)

d8 <-glmmTMB(pmnHCW ~ Wdep + perCC + CIscore + perBOCO + (1|WS/SiteN), data = P1,
REML = FALSE, na.action = na.exclude)

res.d8<-residuals(d8)
qqPlot(res.d8)
shapiro.test(res.d8)

fit.d8 <-fitted(d8)
plot(res.d8 ~ fit.d8)


/comparison of residuals of untransformed vs log(x+1) transformed mean head capsule widths
(lgHCW)

d8a <-glmmTMB(lgHCW ~ Wdep + perCC + CIscore + perBOCO + (1|WS/SiteN), data = P1, REML
= FALSE, na.action = na.exclude)

res.d8a<-residuals(d8a)
qqPlot(res.d8a)
```

shapiro.test(res.d8a)

fit.d8a <-fitted(d8a)
plot(res.d8a ~ fit.d8a)

par(mfrow = c(1, 2))
plot(res.d8 ~ fit.d8)
plot(res.d8a ~ fit.d8a)

/ model residual evaluation for occurrence (pOCR)

d13 <-glmmTMB(pOCR ~ Wdep + perCC + CIscore + perBOCO + (1|WS/SiteN), family = binomial, data = P1, na.action = na.exclude)

*no check normality for pOCR as its binary data consisting of 0s and 1s

res.d13 <-residuals(d13)
fit.d13 <-fitted(d13)
plot(res.d13 ~ fit.d13)

/check overdispersion

*Below code uses Ben Bolker's overdispersion function (estimate - https://bbolker.github.io/mixedmodels-misc/glmmFAQ.html#overdispersion)

```
overdisp_fun <- function(model) {
   rdf <- df.residual(model)
   rp <- residuals(model,type="pearson")
   Pearson.chisq <- sum(rp^2)
   prat <- Pearson.chisq/rdf
   pval <- pchisq(Pearson.chisq, df=rdf, lower.tail=FALSE)
   c(chisq=Pearson.chisq,ratio=prat,rdf=rdf,p=pval)
}
```

overdisp_fun(d13)

//evaluate family specification for density (pDen)

d3a <-glmmTMB(pDen ~ Wdep + perCC + CIscore + perBOCO + (1|WS/SiteN),data = P1, family = gaussian, na.action = na.exclude)
d3b <-glmmTMB(pDen ~ Wdep + perCC + CIscore + perBOCO + (1|WS/SiteN),data = P1, family = poisson, na.action = na.exclude)
d3c <-glmmTMB(pDen ~ Wdep + perCC + CIscore + perBOCO + (1|WS/SiteN),data = P1, family = compois, na.action = na.exclude)
d3d <-glmmTMB(pDen ~ Wdep + perCC + CIscore + perBOCO + (1|WS/SiteN),data = P1, family = nbinom1, na.action = na.exclude)

```
AIC(d3a, d3b, d3c, d3d)

summary(d3a)
summary(d3b)
summary(d3c)
summary(d3d)

res.d3a <-residuals(d3a)
fit.d3a <-fitted(d3a)

res.d3d <-residuals(d3d)
fit.d3d <-fitted(d3d)

par(mfrow = c(1, 2))
plot(res.d3a ~ fit.d3a)
plot(res.d3d ~ fit.d3d)

summary(pDen)


//evaluate addition of zero inflation term for density (pDen)

d3d <-glmmTMB(pDen ~ Wdep + perCC + CIscore + perBOCO + (1|WS/SiteN), data = P1, family =
nbinom1, na.action = na.exclude)
d3dz1 <-glmmTMB(pDen ~ Wdep + perCC + CIscore + perBOCO + (1|WS/SiteN), data = P1, family
= nbinom1, zi = ~1, na.action = na.exclude)
d3dz2 <-glmmTMB(pDen ~ Wdep + perCC + CIscore + perBOCO + (1|WS/SiteN), data = P1, family
= nbinom2, zi = ~1, na.action = na.exclude)

AIC(d3d, d3dz1, d3dz2)

summary(d3d)
summary(d3dz1)

//evaluate zero inflation for occurrence (pOCR)

d13a <-glmmTMB(pOCR ~ Wdep + perCC + CIscore + perBOCO + (1|WS/SiteN), family = binomial,
data = P1, na.action = na.exclude)
d13z1 <-glmmTMB(pOCR ~ Wdep + perCC + CIscore + perBOCO + (1|WS/SiteN), family =
binomial, zi = ~1, data = P1, na.action = na.exclude)

AIC(d13a, d13z1)

summary(d13a)
summary(d13z1)


///run analyses for density (pDen)
```

```
dm1 <-glmmTMB(pDen ~ Wvel + GSScore + Wdep + (1|WS/SiteN), data = P1, family = nbinom1, na.action = na.exclude)
dm2 <-glmmTMB(pDen ~ Wvel + GSScore + SubRich + (1|WS/SiteN), data = P1, family = nbinom1, na.action = na.exclude)
dm3 <-glmmTMB(pDen ~ Wvel + GSScore + perCC + (1|WS/SiteN), data = P1, family = nbinom1, na.action = na.exclude)
dm4 <-glmmTMB(pDen ~ Wvel + GSScore + CIscore + (1|WS/SiteN), data = P1, family = nbinom1, na.action = na.exclude)
dm5 <-glmmTMB(pDen ~ Wvel + GSScore + dCHPm + (1|WS/SiteN), data = P1, family = nbinom1, na.action = na.exclude)

summary(dm1)
summary(dm2)
summary(dm3)
summary(dm4)
summary(dm5)

dmF <-glmmTMB(pDen ~ Wvel + GSScore + SubRich + + CIscore + (1|WS/SiteN), data = P1, family = nbinom1, na.action = na.exclude)
summary(dmF)



///run log(x+1) transformed mean head capsule width (lgHCW) analyses

hm1 <-glmmTMB(lgHCW ~ Wvel + GSScore + Wdep + (1|WS/SiteN), data = P1, REML = FALSE, na.action = na.exclude)
hm2 <-glmmTMB(lgHCW ~ Wvel + GSScore + SubRich + (1|WS/SiteN), data = P1, REML = FALSE, na.action = na.exclude)
hm3 <-glmmTMB(lgHCW ~ Wvel + GSScore + perCC + (1|WS/SiteN), data = P1, REML = FALSE, na.action = na.exclude)
hm4 <-glmmTMB(lgHCW ~ Wvel + GSScore + CIscore + (1|WS/SiteN), data = P1, REML = FALSE, na.action = na.exclude)
hm5 <-glmmTMB(lgHCW ~ Wvel + GSScore + dCHPm + (1|WS/SiteN), data = P1, REML = FALSE, na.action = na.exclude)

summary(hm1)
summary(hm2)
summary(hm3)
summary(hm4)
summary(hm5)

warnings(hm1)
warnings(hm2)
warnings(hm3)
warnings(hm4)
warnings(hm5)
```

* all with convergence problems.

/run with each single variable to see if this resolves convergence issues

hm1a <-glmmTMB(lgHCW ~ Wvel + (1|WS/SiteN), data = P1, REML = FALSE, na.action = na.exclude)
hm2a <-glmmTMB(lgHCW ~ GSScore + (1|WS/SiteN), data = P1, REML = FALSE, na.action = na.exclude)
hm3a <-glmmTMB(lgHCW ~ Wdep + (1|WS/SiteN), data = P1, REML = FALSE, na.action = na.exclude)
hm4a <-glmmTMB(lgHCW ~ SubRich + (1|WS/SiteN), data = P1, REML = FALSE, na.action = na.exclude)
hm5a <-glmmTMB(lgHCW ~ perCC + (1|WS/SiteN), data = P1, REML = FALSE, na.action = na.exclude)
hm6a <-glmmTMB(lgHCW ~ CIscore + (1|WS/SiteN), data = P1, REML = FALSE, na.action = na.exclude)
hm7a <-glmmTMB(lgHCW ~ dCHPm + (1|WS/SiteN), data = P1, REML = FALSE, na.action = na.exclude)

warnings(hm1a)
warnings(hm7a)

*still convergence issues

summary(hm1a)
summary(hm2a)
summary(hm3a)
summary(hm4a)
summary(hm5a)
summary(hm6a)
summary(hm7a)

diagnose(hm7a)

**issue result random effects very small. Drop random effects from model and run

hm1b <-glmmTMB(lgHCW ~ Wvel, data = P1, REML = FALSE, na.action = na.exclude)
hm2b <-glmmTMB(lgHCW ~ GSScore, data = P1, REML = FALSE, na.action = na.exclude)
hm3b <-glmmTMB(lgHCW ~ Wdep, data = P1, REML = FALSE, na.action = na.exclude)
hm4b <-glmmTMB(lgHCW ~ SubRich, data = P1, REML = FALSE, na.action = na.exclude)
hm5b <-glmmTMB(lgHCW ~ perCC, data = P1, REML = FALSE, na.action = na.exclude)
hm6b <-glmmTMB(lgHCW ~ CIscore, data = P1, REML = FALSE, na.action = na.exclude)
hm7b <-glmmTMB(lgHCW ~ dCHPm, data = P1, REML = FALSE, na.action = na.exclude)

warnings(hm1b)
warnings(hm7b)

*warnings still present

diagnose(hm7b)

```
summary(hm1a)
summary(hm2a)
summary(hm3a)
summary(hm4a)
summary(hm5a)
summary(hm6a)
summary(hm7a)

AIC(hm1a, hm1b)
AIC(hm2a, hm2b)
AIC(hm7a, hm7b)

*AIC values the same. no benefit adding random effect

hm5 <-glmmTMB(lgHCW ~ Wvel + GSScore + dCHPm + (1|WS/SiteN), data = P1, REML = FALSE,
na.action = na.exclude)
hm5b <-glmmTMB(lgHCW ~ Wvel + GSScore + dCHPm, data = P1, REML = FALSE, na.action =
na.exclude)

AIC(hm5, hm5b)

*AIC values same no benefit adding random effect

summary(hm5)
summary(hm5b)

*dropping random effect improves model enough to address the convergence issues

diagnose(hm5b)

hm5a <-glmmTMB(lgHCW ~ Wvel + GSScore, data = P1, REML = FALSE, na.action = na.exclude)
hm5ab <-glmmTMB(lgHCW ~ Wvel + GSScore + dCHPm, data = P1, REML = FALSE, na.action =
na.exclude)

anova(hm5a, hm5ab)

ranef(hm5)


hm5 <-glmmTMB(lgHCW ~ Wvel + GSScore + dCHPm + (1|WS/SiteN), data = P1, REML = FALSE,
na.action = na.exclude)
hm5c <-glmmTMB(lgHCW ~ Wvel + GSScore + dCHPm + (1|SiteN), data = P1, REML = FALSE,
na.action = na.exclude)

AIC(hm5, hm5c)


summary(hm5c)
warnings(hm5c)
diagnose(hm5c)
```

/run lgHCW analyses with random effect and without random effect to evaluate if results differ greatly between models with and without random effects

hm1 <-glmmTMB(lgHCW ~ Wvel + GSScore + Wdep + (1|WS/SiteN), data = P1, REML = FALSE, na.action = na.exclude)
hm2 <-glmmTMB(lgHCW ~ Wvel + GSScore + SubRich + (1|WS/SiteN), data = P1, REML = FALSE, na.action = na.exclude)
hm3 <-glmmTMB(lgHCW ~ Wvel + GSScore + perCC + (1|WS/SiteN), data = P1, REML = FALSE, na.action = na.exclude)
hm4 <-glmmTMB(lgHCW ~ Wvel + GSScore + CIscore + (1|WS/SiteN), data = P1, REML = FALSE, na.action = na.exclude)
hm5 <-glmmTMB(lgHCW ~ Wvel + GSScore + dCHPm + (1|WS/SiteN), data = P1, REML = FALSE, na.action = na.exclude)
hmf <-glmmTMB(lgHCW ~ Wvel + GSScore + (1|WS/SiteN), data = P1, REML = FALSE, na.action = na.exclude)

summary(hm1)
summary(hm2)
summary(hm3)
summary(hm4)
summary(hm5)
summary(hmf)

hm1nr <-glmmTMB(lgHCW ~ Wvel + GSScore + Wdep, data = P1, REML = FALSE, na.action = na.exclude)
hm2nr <-glmmTMB(lgHCW ~ Wvel + GSScore + SubRich, data = P1, REML = FALSE, na.action = na.exclude)
hm3nr <-glmmTMB(lgHCW ~ Wvel + GSScore + perCC, data = P1, REML = FALSE, na.action = na.exclude)
hm4nr <-glmmTMB(lgHCW ~ Wvel + GSScore + CIscore, data = P1, REML = FALSE, na.action = na.exclude)
hm5nr <-glmmTMB(lgHCW ~ Wvel + GSScore + dCHPm, data = P1, REML = FALSE, na.action = na.exclude)

hmfnr <-glmmTMB(lgHCW ~ Wvel + GSScore + dCHPm, data = P1, REML = FALSE, na.action = na.exclude)

AIC(hm5, hm5nr)
AIC(hm4, hm4nr)
AIC(hm3, hm3nr)
AIC(hm2, hm2nr)
AIC(hm1, hm1nr)

summary(hm1nr)
summary(hm2nr)
summary(hm3nr)

```
summary(hm4nr)
summary(hm5nr)
summary(hmfnr)


///run occurrence (pOCR) analyses

om1 <-glmmTMB(pOCR ~ Wvel + GSScore + Wdep + (1|WS/SiteN), family = binomial, data = P1,
na.action = na.exclude)
om2 <-glmmTMB(pOCR ~ Wvel + GSScore + SubRich + (1|WS/SiteN), family = binomial, data = P1,
na.action = na.exclude)
om3 <-glmmTMB(pOCR ~ Wvel + GSScore + perCC + (1|WS/SiteN), family = binomial, data = P1,
na.action = na.exclude)
om4 <-glmmTMB(pOCR ~ Wvel + GSScore + CIscore + (1|WS/SiteN), family = binomial, data = P1,
na.action = na.exclude)
om5 <-glmmTMB(pOCR ~ Wvel + GSScore + dCHPm + (1|WS/SiteN), family = binomial, data = P1,
na.action = na.exclude)

summary(om1)
summary(om2)
summary(om3)
summary(om4)
summary(om5)


omF <-glmmTMB(pOCR ~ Wvel + GSScore + SubRich + + CIscore + (1|WS/SiteN), data = P1,
family = binomial, na.action = na.exclude)
summary(omF)
```
